# Using co-occurrence information and trait composition to understand individual plant performance in grassland communities

**DOI:** 10.1038/s41598-018-27017-9

**Published:** 2018-06-13

**Authors:** Eva Breitschwerdt, Ute Jandt, Helge Bruelheide

**Affiliations:** 1 Martin Luther University Halle-Wittenberg, Institute of Biology/Geobotany and Botanical Garden, Am Kirchtor 1, 06108 Halle, Germany; 2 German Centre for Integrative Biodiversity Research (iDiv) Halle-Jena-Leipzig, Deutscher Platz 5e, 04103 Leipzig, Germany

## Abstract

Depending on the strength of environmental filtering and competitive exclusion, successful colonizers of plant communities show varying degrees of similarity to resident species with respect to functional traits. For the present study, colonizer’s performance was assessed in relation to the degree of fit with the resident community, and in addition, in relation to the community’s trait profile and the environmental factors at the study locations. The two-year field experiment investigated the relative growth rates of 130 species that had been transplanted into German grassland communities varying in intensities of land-use. The transplanted species were selected in accordance with the following scenarios: species with highly similar or dissimilar traits to residents, species with highest degree of co-occurrence with resident species and species chosen randomly from the local species pool. The performance of transplanted phytometers depended on the scenario according to which the species were selected, on community trait diversity, and in addition, often on the interaction of both and on land use intensity. The total amount of explained variance in performance was low, but increased considerably when species identity was taken into account. In general, individuals in the co-occurrence scenario performed better than those selected based on trait information or those selected randomly. Different predictors were important in different seasons, demonstrating a limited temporal validity of performance models.

## Introduction

The assessment of trait dispersion patterns of species within communities is commonly used as a tool to understand community assembly mechanisms^1^, with trait requisites being determined by a set of filters constraining colonization, establishment and persistence in a given habitat^2^. While filters are assumed to be mechanistically linked to performance of the individual in the community^3^, few studies have measured the performance of individual plants of a larger number of species along environmental gradients. However, there is also strong evidence that traits affect growth directly and indirectly through biotic interactions. For example, in a transplant experiment conducted in subalpine grasslands hosting five grass species, Gross *et al*. found individual growth to be strongly driven by specific leaf area (SLA)^4^. Similarly, the relative growth rates of 20 common grassland species transplanted into the German biodiversity Exploratory grasslands were best described by the traits of the phytometers^5^.

In grassland communities the strongest filter is often land-use^6,7^. High-intensity land-use in grasslands seeks to increase productivity, involving the extensive application of fertilizer^8^. As a consequence, competition intensity increases with increasing land-use intensity^9^, resulting in a decline in the growth rates of competitively inferior species and increased competitive exclusion^10^. However, such intensive land-use also involves more frequent biomass extraction, either by more frequent mowing or increased stocking densities^11,12^. In consequence, species that are able to regrow after disturbance may be favored^13^, because competition intensity is alleviated^9,14^. Such opposing effects of more intensive land-use make it difficult to predict how any specific plant species responds to simultaneously increased levels of disturbance and nutrient supply. In addition to land-use, species growth strongly depends on climatic conditions. For example, biomass production has been shown to be limited by cold temperatures in spring and high temperatures in combination with low water availability in summer^15^.

Under strong abiotic filtering conditions, species that co-occur in a community are expected to show a high degree of similarity in their functional trait values^16^. In contrast, under competition conditions, those species with less similar traits are more likely to avoid competitive exclusion^10^. In principle, this rule of limiting similarity ensures trait divergence in communities^17^. There is also growing evidence that negative interactions brought about by competition can turn into positive interactions, i.e. facilitation, if the species display trait dissimilarity in certain shared traits. For example, in an experiment on Tibetan grasslands pairwise species interactions became increasingly positive with increasing dissimilarity in maximum height^18^. Similarly, in dry alkali grasslands in Hungary, dissimilarity in canopy height of subordinate species was positively related to the biomass of the dominant species^19^. There are however also limits to trait divergence, as species with extremely diverging trait values might also be excluded as a consequence of strong competition^20,21^, resulting in trait convergence^22^. Furthermore, Gross *et al*. demonstrated that in the same community some traits can show convergence while others exhibit divergence^23^. In consequence, it is not clear whether species that are more similar to a resident community perform better than dissimilar species, or vice versa. In grasslands, this question also depends on land-use intensity. Under heavy land-use intensity, and the associated strong abiotic filtering regime combined with higher competition intensity, newcomers with a higher trait similarity to the extant community should perform better and species with more divergent trait values should perform worse. Recently, we suggested that the optimal degree of trait similarity a new species should have to enter a community can be derived empirically from the probability of co-occurrence with the resident species^24^. These probabilities can be extracted from large vegetation databases (such as the German Vegetation Reference Database, GVRD)^25^ without making any assumptions on trait similarities or dissimilarities between a new species and the receiving community. In our previous paper we found that species that commonly co-occur with the resident species in a community survived best^24^, and accordingly, we also expected them to also perform best.

Finally, under a given level of land-use intensity and filtering conditions, a community itself might determine plant growth. It has convincingly been demonstrated that productivity in grassland communities is positively affected by producer diversity^26^. In particular, biomass production was shown to be higher in communities with higher functional diversity (FD)^27–29^. Such relationships have mostly been based on community responses and rarely tested for individual plant species^30–33^. As the community response is the sum of all individual plant responses, one would expect that, on average, individual plant performance might increase with community FD. In addition to FD, the potential to integrate new species into a community might also depend on the abundance-weighted mean values of certain traits^34^, expressed as community-weighted means (CWM)^35^. For example, a community with taller plants on average might also force new species to grow taller to access enough light. Similarly, plants in a community with low leaf dry matter content (LDMC) tended to show higher growth rates than in those with high LDMC^36^. Such functional attributes of the community are not independent of each other, as FD and CWM can also be the result of external environmental filtering processes, such as land-use intensity and, in turn, may indirectly contribute to environmental filtering themselves. For example, CWMs of SLA have been found to increase with increasing fertilization or disturbance intensity^37–39^, which should result in a high photosynthetic capacity and overall improved growth conditions, thereby intensifying the competition intensity for light.

For the present study, we set out to disentangle the impact of land-use and community trait composition in a large transplant experiment in mesic grasslands differing in land-use intensity and community trait composition. We used extant grassland communities and made use of the given land-use but manipulated the degree of how well a species new to the community might fit into that community. This putative fit was varied by selecting species according to four different scenarios^24^. Two of the scenarios were trait-based, selecting the species most similar and dissimilar to the resident species (“Sim” and “Dissim”). In the third scenario, species were introduced to the community that had the highest degree of co-occurrence with the resident species (“Beals”), while in the fourth scenario species were chosen randomly (“Random”). The objective of our study was to identify the predictors (land-use intensity, CWM or FD of key traits) that determined growth rates and biomass production as well as the actual traits of the colonizing species within the respective grassland communities. We monitored transplants over two years and tested whether the species’ performance differed between seasons. In particular, we hypothesized, (i) that the species with highest probability of occurring in the resident community (i.e. those in the Beals scenario) perform better than those of the other scenarios with respect to growth rates and biomass production under all conditions of land-use intensity and community trait composition. (ii) Furthermore, we expected species similar to the resident species to perform better with increasing land-use intensity. (iii) Moreover, of all drivers of plant performance investigated we hypothesized land-use intensity to have a higher explanatory power on species growth and biomass production than FDs and CWMs. (iv) Finally, we tested whether in addition to the selection scenario, land-use intensity and community trait composition, climate (including air and soil temperature, relative air humidity and soil moisture) had additional impact on the phytometers’ responses.

## Results

**Figure 1:**
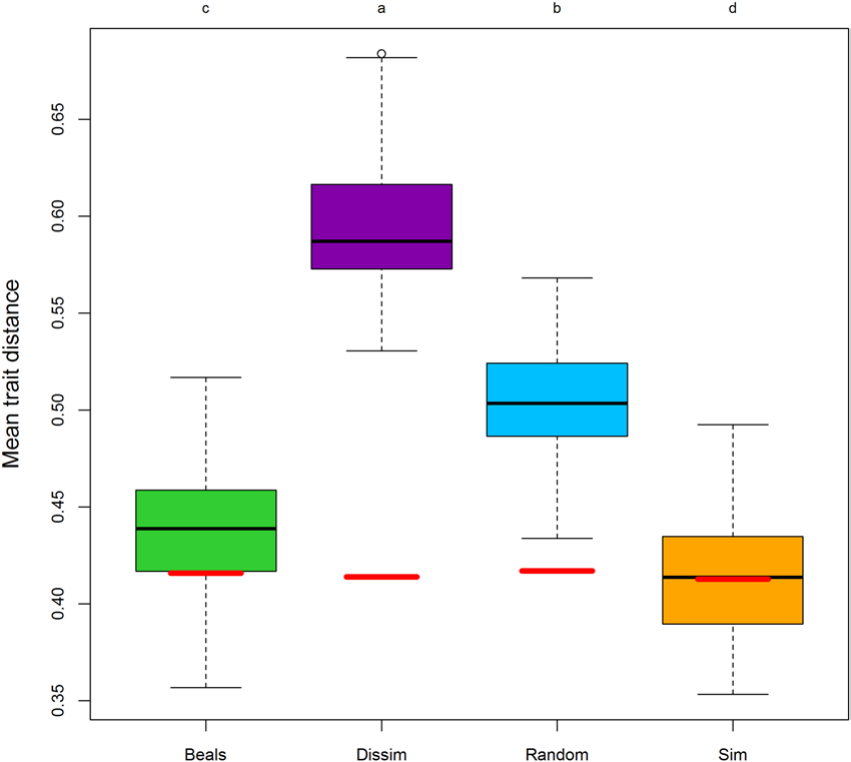
Mean pairwise trait distances between the six introduced species in the four scenarios (Beals, Dissim, Random and Sim) and all resident species. Values are multi-trait distances and based on eight traits. Boxes show quartiles and medians across all 54 plots and two subplots per plot (n = 108 per scenario). Whiskers show 1.5 times the interquartile ranges. Small letters indicate statistically significant differences among the scenarios according to a Tukey post-hoc test. As a reference, the red lines show the mean pairwise trait distances among the resident species before six phytometer species were planted into every subplot.

Before the six phytometer species were planted into the plots, mean multi-trait distance did not differ among the resident species that grew in the respective subplots, which had been randomly assigned to the four different scenarios (Fig. 1). While the colonizer species in the Sim scenario displayed exactly the same trait dissimilarity to residents as the residents did among themselves, (with a multi-trait dissimilarity of 0.45), the species in all other scenarios were more dissimilar to the resident species in the respective subplots, with 0.47, 0.54 and 0.64 in the Beals, Random and Dissim scenarios, respectively.

**Figure 2:**
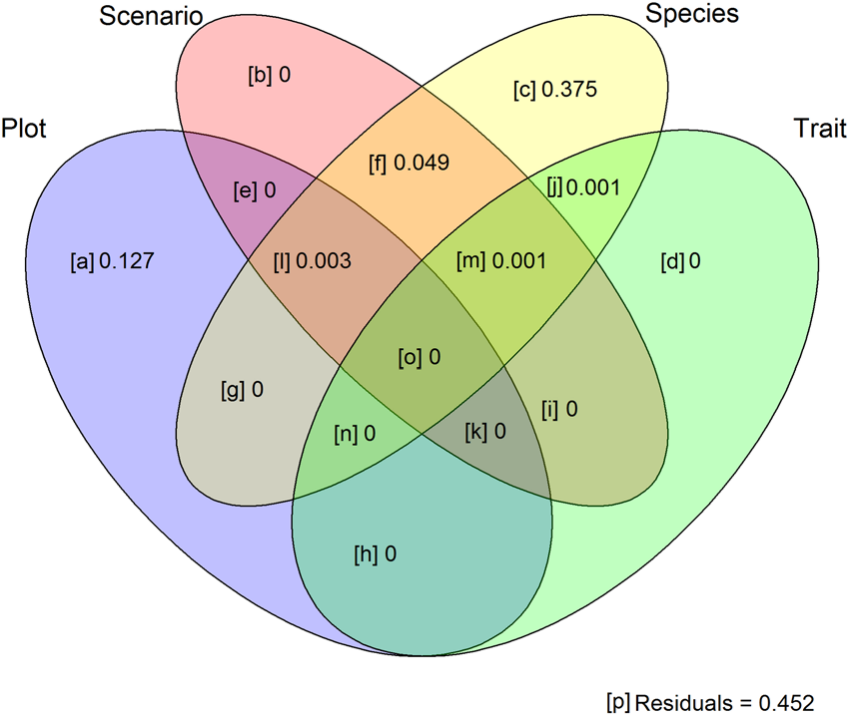
Variance partitioning for RGR height of the first vegetation period 2012 (interval 1–4) correlated with FD of SLA. Results for all other response variables are given in SI Table S4. Variance components <0.001 not shown.

The design variables in our study - i.e. the identity of the species planted into the plots, the identity of the plot and the scenario of how the species were selected - explained a varying overall amount of variation in the responses (Table 1). Variation explained by plot ranged from 4% for RGR of leaf number in the first monitoring interval to 39% for RGR height in the 3rd interval, between 0% and 0.4% explained by scenario (for RGR leaf length 1st interval) and between 9% (for RGR leaf length 3rd interval) and 38% (for RGR height 1st interval) explained by species identity. As an example, Fig. 2 shows the variances in RGR of height of the first monitoring interval exclusively and jointly explained by plot, scenario, species and trait variable (SLA FD). In the variance partitioning analysis, community traits explained maximally 6.5% (CWM of SLA for aboveground biomass at harvest, SI Table S4). Whenever trait variables explained variance, this fraction was also jointly explained by plot and species but not by scenario (column n in SI Table S4 compared to columns i, k, o, m, see also Fig. 2), which indicate that community trait variables did not vary much with environmental differences among subplots.

**Table 1:** Proportional variance of RGR of all variables at all observation intervals (1–4 = vegetation period 2012; 4–5 = winter 2012/2013; and 5–7 = vegetation period 2013) and aboveground biomass, LDMC and SLA at the final harvest in September 2013), exclusively explained by plot, scenario and species, jointly by two of these factors or all of them as well as residual variance.

Response variable	Exclusively explained by			Jointly explained by				
	Plot	Scenario	Species	Plot & Scenario	Scenario & Species	Plot & Species	Species, Plot & Scenario	Residual Variance
RGR height 1–4	0.127	0	0.376	0	0.05	0	0.002	0.452
RGR p. proj. area 1–4	0.121	0	0.203	0	0	0.006	0	0.67
RGR leaf length 1–4	0.127	0.003	0.206	0	0.004	0.002	0.002	0.658
RGR leaf number 1–4	0.043	0.002	0.202	0	0.009	0.014	0	0.731
RGR height 4–5	0.356	0.001	0.146	0.001	0.033	0	0	0.478
RGR p. proj. area 4–5	0.204	0	0.134	0	0.049	0.012	0	0.611
RGR leaf length 4–5	0.275	0	0.106	0	0.025	0.037	0	0.565
RGR leaf number 4–5	0.092	0	0.202	0.001	0.019	0.053	0	0.642
RGR height 5–7	0.391	0.002	0.16	0.004	0.016	0.015	0	0.422
RGR p. proj. area 5–7	0.188	0	0.159	0.001	0.002	0	0	0.657
RGR leaf length 5–7	0.312	0	0.097	0	0.002	0.051	0.002	0.537
RGR leaf number 5–7	0.083	0	0.193	0	0.007	0.012	0	0.71
Biomass	0.121	0	0.222	0.001	0.001	0.061	0.001	0.594
LDMC	0.134	0.002	0.299	0	0.014	0.043	0.017	0.492
SLA	0.191	0	0.224	0	0.024	0.015	0.026	0.522

All components add up to 1. p. proj. area = plant projection area.

The different responses of RGR in the different monitoring periods as well as aboveground biomass, SLA and LDMC at the final harvest were explained to varying degrees by the final mixed linear regression models (Table 2). The conditional R² captured by these models explained between 33% and 68% variation, while the models’ marginal R² accounted for only 0.4% to 7% variation (Table 2). The difference between conditional and marginal R² showed that random factors significantly contributed to explaining variance, with species identity explaining most (between 9% and 38%), followed by Exploratory (0% to 38%), plot (5% to 17%), and subplot (0% to 4%, SI Table S5).

**Table 2:** Results of the minimum linear mixed effects models for the transplant’s relative growth rates in height, plant projection area, leaf length and number of leaves for the three monitoring periods in 2012 and 2013 and for aboveground biomass, specific leaf area (SLA) and leaf dry matter content (LDMC) at the final harvest in September 2013.

Responses	Predictors	Estimate	p-value	Marginal R²	Conditional R²
**Veg. period 2012**					
RGR height	Intercept	0.150	0.424	0.004	0.536
	Multi-trait FD	0.058	0.038		
RGR p. proj. area	Intercept	−0.018	0.865	0.008	0.419
	Multi-trait FD	0.097	0.004		
RGR leaf length	Intercept (Scen Beals)	0.085	0.551	0.011	0.384
	Height FD	0.068	0.024		
	Scen Dissim	−0.094	0.411		
	Scen Random	−0.225	0.005		
	Scen Sim	−0.028	0.704		
RGR leaf number	Intercept (Scen Beals)	0.129	0.216	0.013	0.328
	SLA FD (Scen Beals)	0.097	0.050		
	Scen Dissim	−0.154	0.202		
	Scen Random	−0.262	0.002		
	Scen Sim	−0.154	0.052		
	SLA FD:Scen Dissim	−0.102	0.126		
	SLA FD:Scen Random	−0.236	0.002		
	SLA FD:Scen Sim	−0.069	0.318		
**Winter 2012/2013**					
RGR height	Intercept (Scen Beals)	−0.039	0.918	0.069	0.634
	LUI	0.250	0.000		
	Height FD (Scen Beals)	0.137	0.006		
	Height CWM (Scen Beals)	−0.142	0.025		
	Scen Dissim	−0.163	0.107		
	Scen Random	−0.194	0.009		
	Scen Sim	0.065	0.340		
	Height FD:Scen Dissim	−0.178	0.013		
	Height FD:Scen Random	−0.070	0.345		
	Height FD:Scen Sim	−0.199	0.007		
	Height CWM:Scen Dissim	0.171	0.020		
	Height CWM:Scen Random	0.024	0.726		
	Height CWM:Scen Sim	0.173	0.020		
RGR p. proj. area	Intercept (Scen Beals)	0.014	0.957	0.061	0.492
	LUI	0.215	0.001		
	Height FD (Scen Beals)	0.183	0.000		
	SLA CWM	0.130	0.006		
	LDMC CWM	0.092	0.035		
	Height CWM	−0.100	0.035		
	Scen Dissim	−0.194	0.095		
	Scen Random	−0.185	0.025		
	Scen Sim	−0.054	0.468		
	Height FD:Scen Dissim	−0.146	0.027		
	Height FD:Scen Random	−0.173	0.013		
	Height FD:Scen Sim	−0.153	0.026		
RGR leaf length	Intercept (Scen Beals)	0.060	0.863	0.051	0.544
	LUI	0.210	0.001		
	Height FD (Scen Beals)	0.129	0.016		
	Height CWM (Scen Beals)	−0.119	0.077		
	Scen Dissim	−0.218	0.039		
	Scen Random	−0.091	0.249		
	Scen Sim	0.001	0.984		
	Height FD:Scen Dissim	−0.156	0.045		
	Height FD:Scen Random	−0.079	0.326		
	Height FD:Scen Sim	−0.213	0.008		
	Height CWM:Scen Dissim	0.133	0.094		
	Height CWM:Scen Random	0.041	0.586		
	Height CWM:Scen Sim	0.210	0.009		
RGR leaf number	Intercept (Scen Beals)	−0.168	0.297	0.013	0.440
	LUI	0.086	0.086		
	Height FD	0.057	0.075		
	SLA CWM (Scen Beals)	0.024	0.659		
	Scen Dissim	0.016	0.904		
	Scen Random	0.008	0.927		
	Scen Sim	0.045	0.554		
	SLA CWM:Scen Dissim	−0.093	0.155		
	SLA CWM:Scen Random	−0.068	0.308		
	SLA CWM:Scen Sim	0.066	0.269		
**Veg. period 2013**					
RGR height	Intercept	0.079	0.864	0.012	0.682
	LUI	−0.126	0.046		
RGR p. proj. area	Intercept	0.029	0.910	0.005	0.398
	SLA FD	0.086	0.020		
RGR leaf length	Intercept	0.017	0.967	0.026	0.568
	SLA FD	0.124	0.000		
	Height CWM	−0.138	0.001		
RGR leaf number	Intercept	0.040	0.729	0.014	0.357
	Multi-trait FD	−0.090	0.017		
	SLA FD	0.114	0.005		
**Harvest 2013**					
Biomass	Intercept (Scen Beals)	−0.016	0.865	0.064	0.423
	LUI	0.170	0.002		
	Multi-trait FD (Scen Beals)	0.003	0.960		
	LDMC FD (Scen Beals)	−0.116	0.076		
	SLA CWM (Scen Beals)	−0.094	0.107		
	Scen Dissim	−0.116	0.382		
	Scen Random	−0.066	0.476		
	Scen Sim	−0.048	0.535		
	Multi-trait FD:Scen Dissim	0.217	0.029		
	Multi-trait FD:Scen Random	0.064	0.514		
	Multi-trait FD:Scen Sim	−0.080	0.333		
	LDMC FD:Scen Dissim	0.052	0.581		
	LDMC FD:Scen Random	0.018	0.854		
	LDMC FD:Scen Sim	0.226	0.008		
	SLA CWM:Scen Dissim	0.292	0.000		
	SLA CWM:Scen Random	0.228	0.007		
	SLA CWM:Scen Sim	0.185	0.016		
SLA	Intercept (Scen Beals)	0.095	0.683	0.044	0.509
	Height CWM	0.140	0.001		
	LDMC CWM (Scen Beals)	−0.118	0.024		
	Scen Dissim	−0.347	0.005		
	Scen Random	−0.040	0.644		
	Scen Sim	0.041	0.574		
	LDMC CWM:Scen Dissim	0.196	0.003		
	LDMC CWM:Scen Random	0.212	0.003		
	LDMC CWM:Scen Sim	−0.013	0.825		
LDMC	Intercept (Scen Beals)	0.062	0.557	0.036	0.556
	SLA FD	−0.080	0.030		
	LDMC FD	0.077	0.042		
	SLA CWM (Scen Beals)	−0.020	0.757		
	LDMC CWM (Scen Beals)	0.065	0.300		
	Scen Dissim	0.275	0.041		
	Scen Random	0.037	0.682		
	Scen Sim	0.046	0.531		
	SLA CWM:Scen Dissim	−0.005	0.952		
	SLA CWM:Scen Random	−0.076	0.362		
	SLA CWM:Scen Sim	0.164	0.027		
	LDMC CWM:Scen Dissim	−0.041	0.611		
	LDMC CWM:Scen Random	−0.268	0.001		
	LDMC CWM:Scen Sim	0.104	0.157		

All models were simplified starting with the same suite of predictors: land-use intensity (LUI), community weighted means (CWM), functional diversity (FD), scenario (Beals, Dissim, Random, Sim, see text for explanation) and all interactions of scenario with LUI, CWM, and FD. CWM and FD were based on the single traits SLA, LDMC and height, while multi-trait FD was based on all eight traits (see Methods). All variables were scaled by mean and standard deviation, thus the estimates show the direction and magnitude of impact on the plant responses. Marginal R^2^ refers to the variance explained by fixed factors and conditional R^2^ to the variance explained by both fixed and random factors. Random factors in the model included Exploratory (Schwäbische Alb, Hainich and Schorfheide), plot (n = 54) nested in Exploratory, subplot (n = 432) nested in plot and species identity (n = 130). For variance of random factors see SI Table S5.

**Figure 3:**
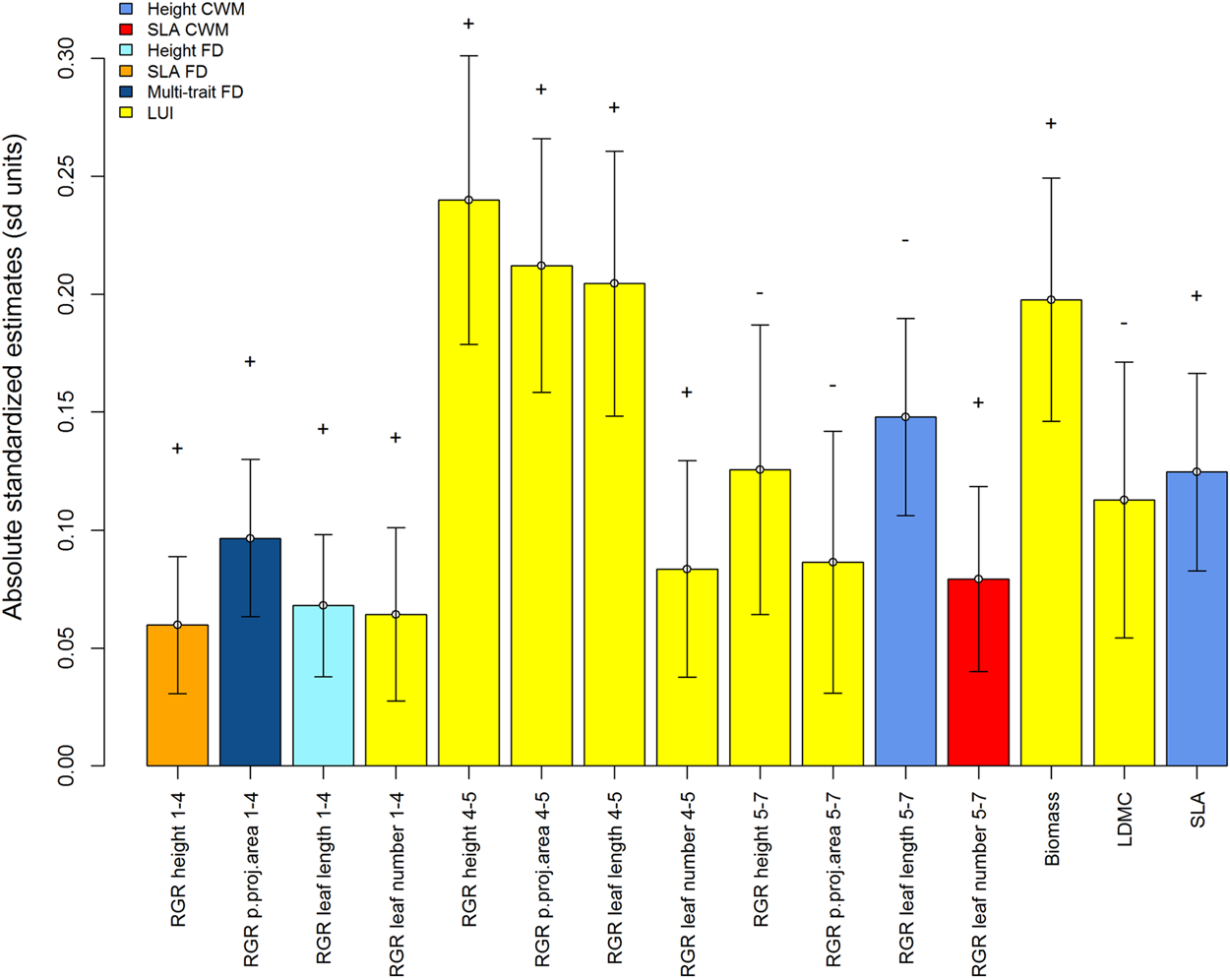
Absolute standardized model estimates of the best single-predictor models with their corresponding standard errors for the different growth rates (RGR of height, plant projection area, leaf length and leaf number) in the three time intervals (1–4, 4–5 and 5–7), and aboveground biomass, LDMC and SLA at the time of the final harvest. Only the predictors (see different color legend) are shown that had the highest explanatory power on the responses. Multi-trait FD refers to FD based on eight traits (SLA, LDMC, height, leaf anatomy, leaf persistence, leaf distribution, physical defense and vegetative reproduction). Plus and minus signs above bars indicate positive or negative effects.

The best single-predictor mixed models revealed different predictors for the different observation intervals, with LUI occurring in most of the best models, and with positive and negative estimates on growth variables in the winter and second summer intervals, respectively (Fig. 3). CWMs of SLA and height affected RGRs only in the second summer intervals, while FD measures were only the best predictors in the first vegetation period, with FD of SLA, FD of height and multi-trait FD positively affecting RGR in height, leaf length and plant projection area, respectively (Fig. 3).

**Figure 4:**
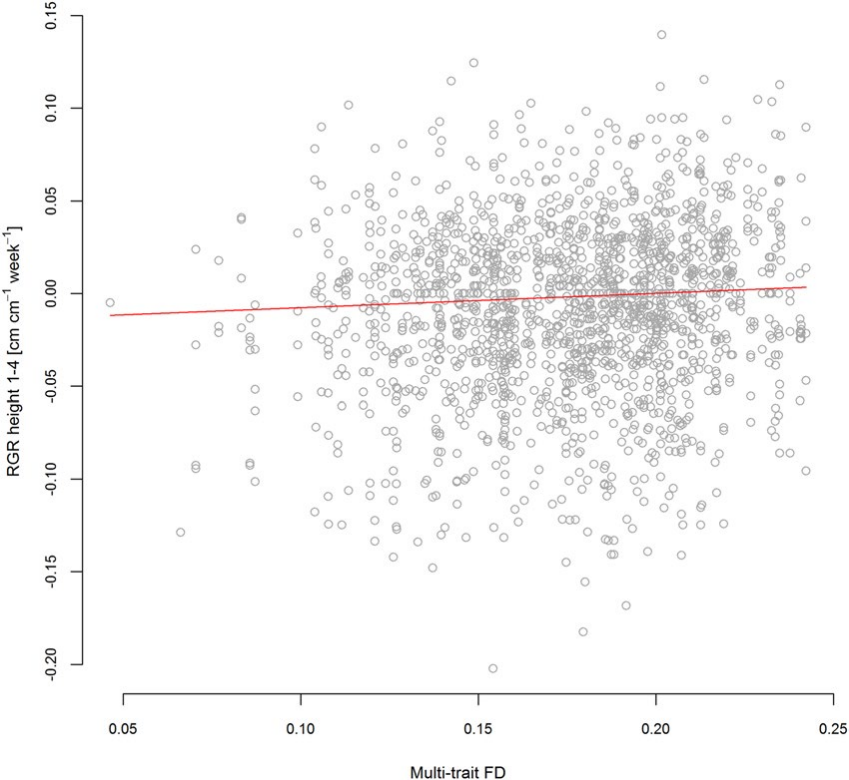
RGR height in the first vegetation period (1–4) as a function of Multi-trait FD. For parameter estimates and p-values see Table 2; for variance of random factors see SI Table S5.

**Figure 5:**
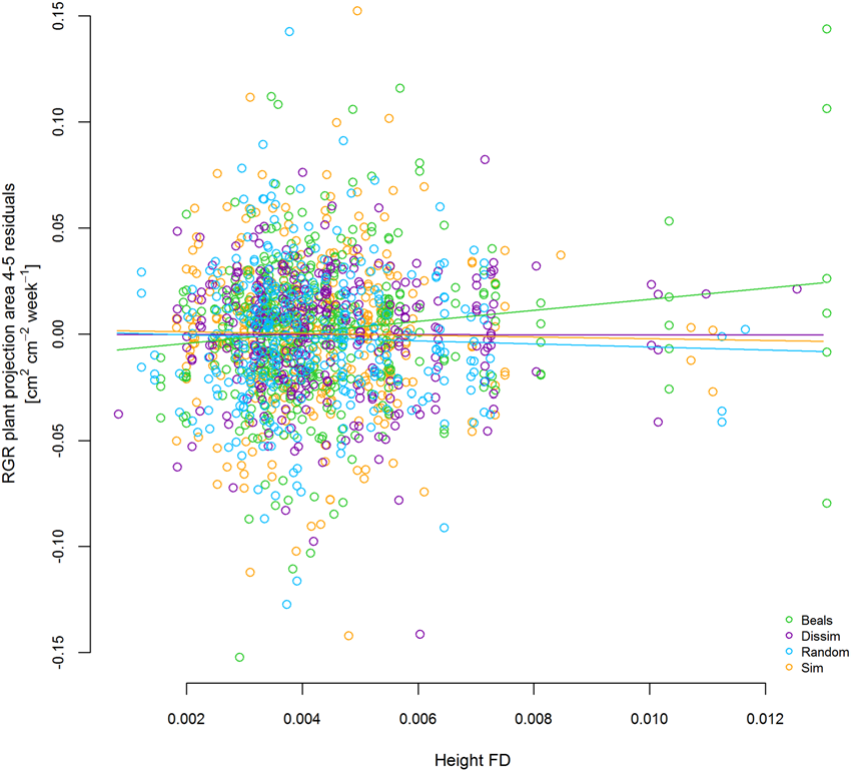
RGR plant projection area residuals in winter (4–5) as a function of height FD and scenario. For parameter estimates and p-values see Table 2; for variance of random factors see SI Table S5.

**Figure 6:**
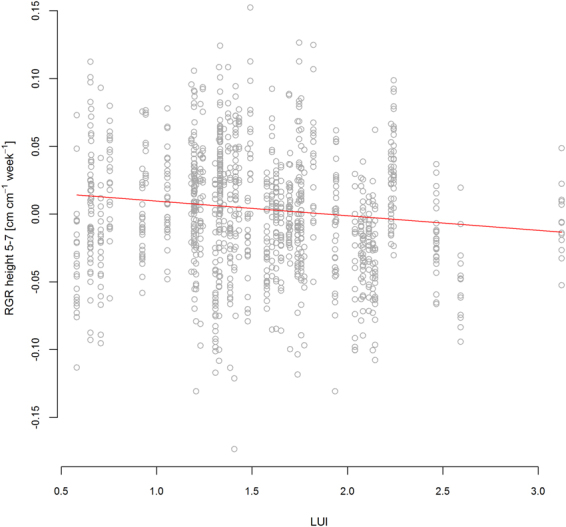
RGR height in the second vegetation period (5–7) as a function of LUI. For parameter estimates and p-values see Table 2; for variance of random factors see SI Table S5.

**Figure 7:**
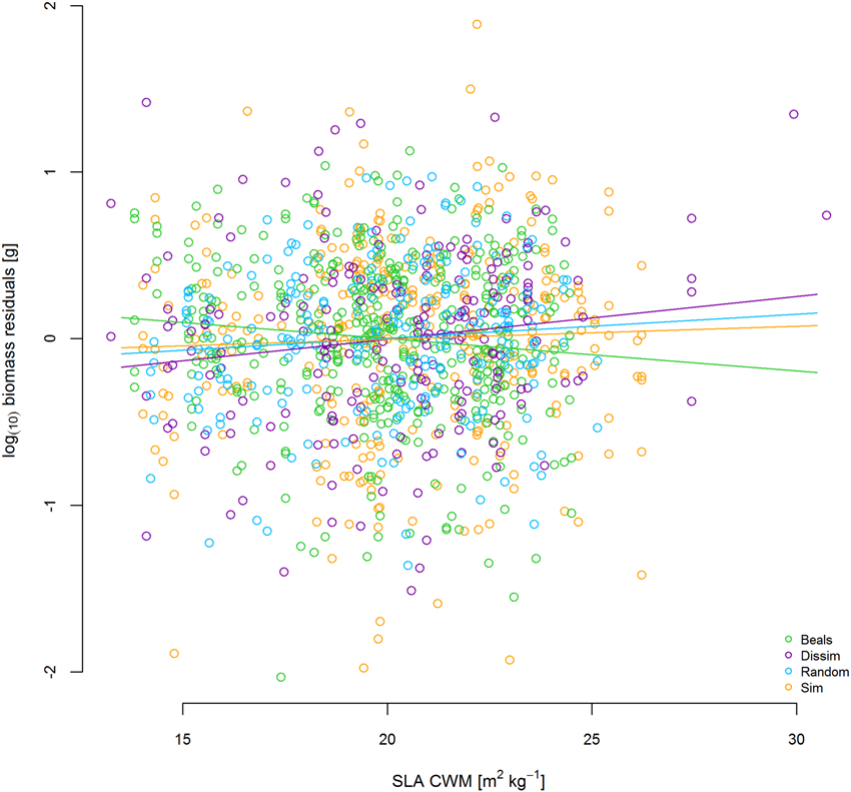
Aboveground biomass residuals (log scale) at time of harvest (end of second vegetation period) as a function of SLA CWM and scenario. For parameter estimates and p-values see Table 2; for variance of random factors see SI Table S5.

**Figure 8:**
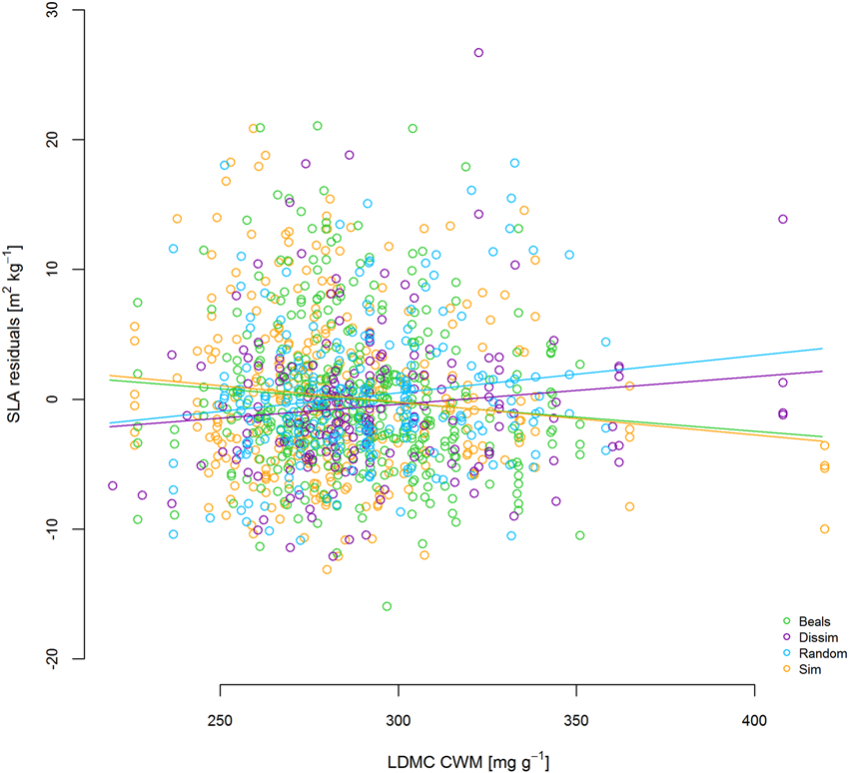
SLA residuals at time of harvest (end of second vegetation period) as a function of LDMC CWM and scenario. For parameter estimates and p-values see Table 2; for variance of random factors see SI Table S5.

In many cases, the optimized multi-predictor models identified the same predictors as shown in the single predictor models of Fig. 3. For example, in the vegetation period in 2012 multi-trait FD was the sole predictor for RGR in plant projection area and FD of height predicted RGR of leaf length (Table 2). Similarly, the best models for RGR in height in winter and early spring (2012/2013) contained LUI as a predictor (Table 2). In the same monitoring intervals, the different growth variables were best explained by different predictors. For example, the best predictors for RGR in height and projection area in the first monitoring interval were multi-trait FD (Fig. 4, Table 2), while leaf length and leaf number were best predicted by FD of height or SLA in combination with scenario (Table 2). In the winter monitoring interval, plant performance depended strongly on FD (Fig. 5). In the same period, LUI was a predictor in all significant models (Table 2) and remained important the following summer and at the final harvest (Table 2, Fig. 6). In general, growth rates increased with LUI in winter, but decreased with LUI in the subsequent summer (Figs. 3, 6, Table 2). At the final harvest, aboveground biomass was again positively related to LUI (Table 2). Across all models, scenario was a more frequent predictor than LUI and occurred in nine of the 15 models. Seven of these nine models predicted performance, of which five models displayed highest growth rates of phytometers in the Beals scenario, followed by Sim, while Dissim and Random ranked lowest (Table 2). Plants in the Beals scenario also performed better in combination with trait measures, such as with FD of height (Fig. 5, increasing RGR of plant projection area in the Beals scenario in winter). Growth of plants in the Beals scenario also depended differently on traits. At the final harvest, in contrast to the other three scenarios aboveground biomass decreased with increasing CWM of SLA (Fig. 7). Similarly, interactions with scenario were encountered in the explaining of SLA (Fig. 8) and LDMC (Table 2) at the final harvest. In general, the responses of transplants in the Beals scenario often differed from those in the other scenarios, particularly when compared to those in the Random and Dissim scenarios (Figs. 5, 7 and 8). In contrast, the patterns in the Sim scenario were sometimes closer to the Beals scenario (Fig. 8) or the Random and Dissim scenarios (Figs. 7 and 5). Among the remaining predictors, FD explained growth in more of the models than CWM. FD was retained in 13 of the 15 models across all monitoring intervals, while CWM was only retained in eight of them (Table 2). Among all FD measures, multi-trait FD, on which the species selection for the scenarios was based, FD of SLA and FD of height were the most frequent predictors for plant performance and were included in four, five and five models, respectively (Table 2). In contrast, CWM was more important in explaining the community mean SLA (Fig. 8) and LDMC (Table 2) at the time of harvest.

**Figure 9:**
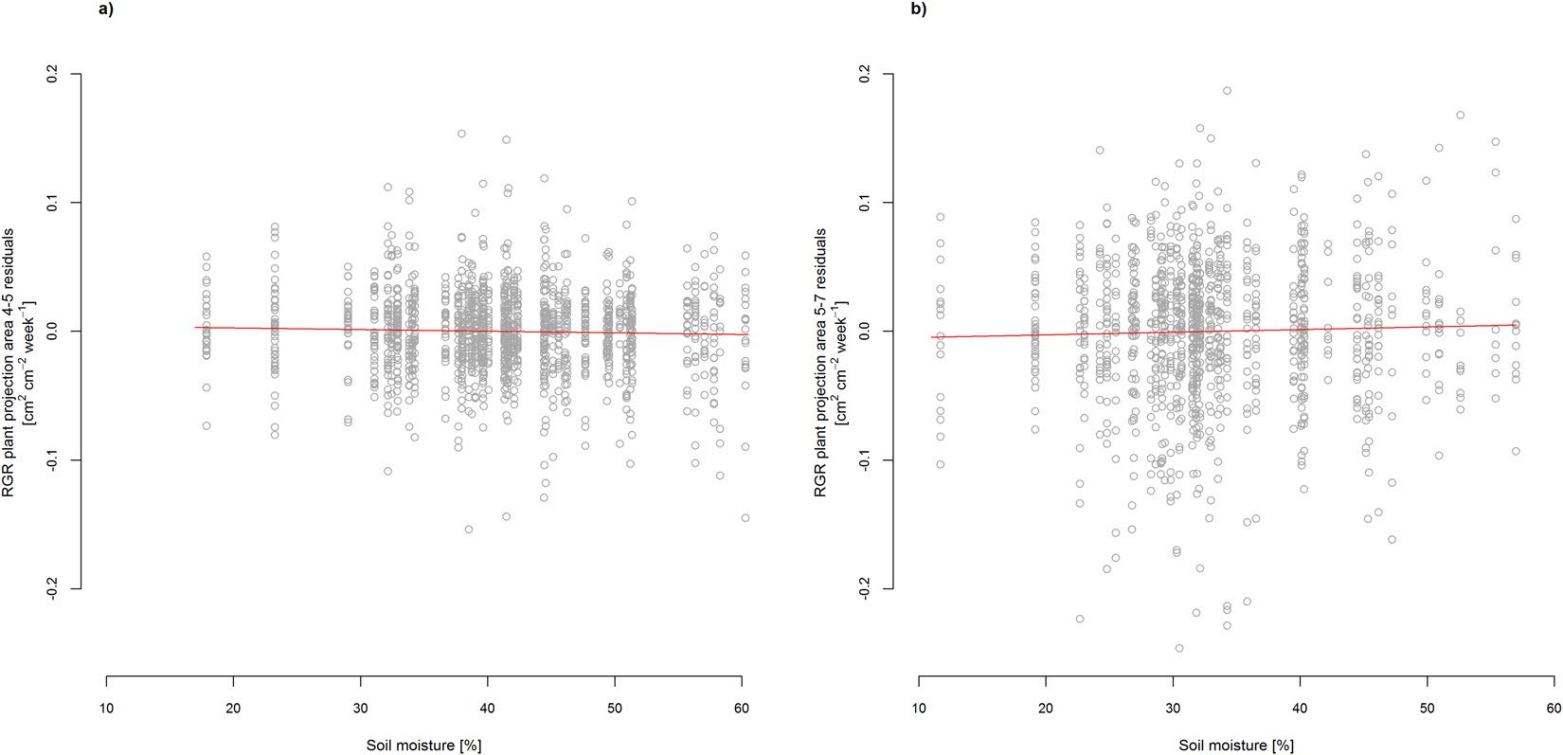
RGR plant projection area residuals (**a**) in winter 2012/2013 (4–5) and (**b**) in the following vegetation period 2013 (5–7) as a function of soil moisture. For parameter estimates and p-values see SI Table S6.

The climatic conditions differed significantly between the two summer periods and the winter with respect to relative air humidity and soil moisture (SI Fig. S2). The second vegetation period in 2013 was also warmer, as demonstrated by higher air and soil temperatures. Adding each one of these four climate variables to the final models did not result in model improvement, with the exception of soil moisture (SI Table S6). In winter, soil moisture had significantly negative impacts on growth variables, while in summer the effects were positive, e.g. on RGR plant projection area (Fig. 9, SI Table S6). In addition, soil moisture had also positive effects on RGR of height and leaf length in the vegetation period 2013 and on SLA and LDMC at the time of harvest (SI Table S6). In all cases, where models were improved by including climate variables, the other predictors remained significant after the climate variable had been added.

## Discussion

We showed that the performance of newly colonizing species strongly depended on the trait composition of the resident community and land-use intensity. In addition, the scenario according to which the phytometer species were selected had a strong influence on how well the phytometer performed.

In many of the monitoring intervals and for many growth variables, phytometer species selected by the scenario based on co-occurrence probability of added and resident species derived from a vegetation database (Beals) performed better than those selected by trait information (Sim, Dissim) or random selection (Random). These results support our first hypothesis and also confirm the observed higher survival rates in the Beals scenario^24^. More generally, this finding demonstrates the huge potential of co-occurrence-based approaches in growth models^24,40^. Although no traits were used in the Beals selection process of species, and the species selected were not as similar as they possibly could have been, a surprising feature of this scenario was that the added species did not result in a change in mean pair-wise trait distances across all plots^24^. Assuming the species in the resident community had a trait composition filtered by land-use and other factors at that site, and that this trait composition facilitated the survival of the resident species, the traits of the newcomer species in the Beals scenario had exactly the same degree of similarity or dissimilarity to the resident species, which increased their survival and, at the same time, their performance compared to species in the other scenarios. This also implies that rather than the most similar or dissimilar species, it were those with trait values at intermediate distances to the resident species that performed best. The Beals scenario was found to rank closer to the Sim than to Dissim scenario (at 32% of the distance between Sim and Dissim)^24^. This also explains why species in the Sim scenario often ranked second in growth after those in the Beals scenario but were superior to the Dissim and Random scenarios. It is however noted that the phytometer species of the Beals scenario did not always show the highest growth rates in all intervals or the highest aboveground biomass production. Slow growth, shade tolerance, higher investment into roots compared to leaves or other strategies alternative to fast growth^41^ might also apply to the higher survival success of species of the Beals scenario^24^. Similarly, our second hypothesis has to be rejected that species of the Sim scenario performed better with increasing land-use intensity because none of the best models included the interaction between land-use and scenario.

As stated in our third hypothesis, land-use intensity was one of the strongest drivers of phytometer performance. In our study, LUI often had an additive effect together with scenario, FD and CWM on the growth of plant individuals, particularly in the winter and early spring interval. In this period, fewer disturbances occurred and the plants had the chance to grow without being eaten, cut or trampled on. As a high LUI is often combined with high levels of fertilization, early spring was probably the season when plants benefitted most from a higher nutrient supply. In contrast, LUI had a negative effect on height growth in the subsequent summer interval, probably because the plants remained smaller due to more frequent mowing or grazing events.

In addition to the strong effect of scenario and land-use intensity, and often interacting with these predictors, FD also played a role in the performance of the added species, which confirms other approaches of predicting biomass from traits^42^. With the exception of trait responses (SLA and LDMC at the time of harvest), the estimates for FD measures on growth were always positive, showing that the added species benefitted from a functionally more diverse community. In particular, FD in SLA was found to be a consistent positive predictor. Given that SLA reflects the main axis in the leaf economics spectrum^43^, the importance of FD of SLA points to a pattern of niche partitioning in resource use. For example, it has been described that grassland species in diverse mixtures absorb up to 20% more light than those in monocultures as a result of a greater three-dimensional use of space, brought about by more overlapping plant architecture, and in consequence, a higher biomass density^44^. Consistent with our results, the authors also encountered an increase in canopy height^44^. Similarly, a positive correlation between individual plant height and functional richness^45^ and an increased aboveground use of space with increasing functional richness^46^ is in accordance with our results. Some FD predictors only had effects in certain scenarios, such as FD of height, which increased the growth rates in plant area in the Beals scenario only. Thus, it might well be that FD effects can only play out if the species added to the community have already been environmentally filtered. Then, the finding that multi-trait FD, which was based on the traits chosen by us for devising the scenarios, was also a frequent predictor is an indication that the traits chosen for this index are ecologically meaningful for growth and persistence.

Finally, there were also a few but notable effects of CWM trait values on plant performance. For example, CWM of SLA had a positive impact on aboveground biomass at the end of the experiment in the Sim, Dissim and Random scenario in the second year, which might reflect better overall resource supply. However, the significant interaction with scenario and the negative response of species in the Beals scenario shows that different species respond differently to a community’s trait composition, and the conditions that are favorable for one group might be disadvantageous for another. For example, a high CWM of SLA also indicates stronger competition for light^47^, making it more difficult for less tall species to persist in the shady undergrowth^48^. However, the increased resource partitioning of light through the addition of smaller species to a community, can result in only very slight increases in community biomass production^49^. Another explanation for a negative response to CWM of SLA might be that the FD of a trait is not independent from the CWM of the same trait, since trait variation is constrained by the mean^50^. Thus, extreme values of CWM values result in low values of FD and conversely, a negative relationship to CWM of a trait might only indicate a positive relationship to FD of the same trait. Finally, responses to CWM also depended on the different scenarios. Accordingly, the expected negative relationship of the target plant’s SLA with the CWM of LDMC^51^ was only encountered in the Sim and Beals scenarios, where species had been selected with the highest similarity (Sim) or at least with some similarity (Beals) to the resident community. The finding that the target plant’s SLA increased with the CWM of LDMC in the Random and Dissim scenarios shows that they became more divergent to the residents, which simply reflects the selection procedure.

Despite the clear patterns found in our study, a lot of variation in growth remained unaccounted for. The high importance of random factors such as “Exploratory” and “plot” results from the realistic field conditions under which the experiment has been carried out. The vast range of soil, climate and management conditions across all plots were only partially captured by the few environmental variables used as fixed predictors in our study. Similarly, the high variation brought about by species identity is explained by the large pool of species (130) from which we drew the phytometer species for the different scenarios. We also may have missed an important compartment of the plants. As we only focused on aboveground biomass we do not know whether allocation patterns between leaves and roots differed among scenarios. For example, in another study on the same grasslands, root volume was found to increase with land-use intensity and root-to-shoot ratio to depend both on the local neighbourhood and the level of land-use intensity^52^. However, they found root biomass to be only poorly predicted by traits and environmental factors^5^. In contrast to our study, where the phytometer traits were captured in the scenarios, Herz *et al*. used single traits measured on the phytometers to predict performance. In their models, root traits such as root calcium and root carbon content considerably improved the model quality for aboveground biomass. Root carbon concentration indicates the prevalence of more reduced and polymerized structural carbohydrates, which was negatively related to above- and belowground growth^5^. However, accounting for root traits in our study would have required to include them in the different scenarios from the beginning, which was not feasible given the large size of the species pool from which the scenarios were constructed. Elsewhere, in the high semi-arid Andes, it has been observed that with increasing grazing pressure, more biomass is allocated to roots^53^. Similarly, we do not know the proportion of aboveground biomass extracted by land-use. However, biomass measurements in the Exploratory grassland plots have shown that productivity increased with higher levels of fertilization^8^ and fertilization is a component of the LUI^9^. We therefore have to acknowledge that monitoring growth rates with simultaneous biomass extraction does not allow for simple explanations. Many plant individuals had more or less the same aboveground size at the end of our experiment as at the beginning. As grazing occurred at different times in different plots, and compensatory growth after grazing also varies with time of recovery^54^, the fixed monitoring dates might not have always captured plant growth in the most accurate way. However, given the logistic effort already involved, plot-wise adaptation of monitoring dates would not have been feasible. Such varying dates would also have precluded relationships to weather conditions, which varied over time and space. In addition, incorporating climate variables into our models supported our fourth hypothesis that they explained additional variance. In particular, soil moisture had a positive effect on plant growth and SLA in summer 2013, when temperatures were higher than in summer 2012. This is in accordance with findings of increased biomass production and growth at increased soil moisture under warm conditions^55–57^. In contrast, under low temperatures in winter soil moisture had a negative effect on plant growth, which might be explained by water logging which negatively affects N mineralization^58^. Accordingly, seasonal variation in RGR, biomass production and SLA has also been reported in numerous other studies (e.g.^59–61^).

In summary, our finding that co-occurrence information allows conclusions to be drawn on plant growth bodes well for the capability to predict individual as well as community performance from vegetation databases, which has, as yet, not been attempted before. This potential predictive power became particularly evident when we combined co-occurrence data with functional traits. The fact that plant responses to community trait composition differed among scenarios, might point to a hierarchical cascade of community assembly. Thus, species might only respond to community FD or CWM after they have passed other environmental filters. We also confirmed the key role of land-use intensity for plant performance^62^. However, the finding that land-use intensity played different roles at different times of the year demonstrates that temporal resolution is required when assessing land-use impacts on plant performance at larger spatial scales^63,64^.

## Materials and Methods

### Study Sites and Experimental Design

We planted different vascular plant species into 54 grasslands communities, making use of the network of experimental plots in the German Biodiversity Exploratories^65^. In each of the three study regions (Schwäbische Alb, South Germany; Hainich, Central Germany and Schorfheide, Northeast Germany), 18 grassland plots were selected that represent the three main land-use types (i.e. each six plots of meadows, pastures and mown pastures). The plots differed in land-use intensity, which was assessed by an index (LUI) that combines mowing and grazing frequencies, number of grazers per hectare and fertilization levels^9^ according to formula (1).

1 $$LU{I}_{p}=\sqrt{\frac{{G}_{p}}{\bar{G}}+\frac{{F}_{p}}{\bar{F}}+\frac{{M}_{p}}{\bar{M}}}$$

The land-use index for a site *p* (*LUI*_*p*_) was calculated from the sum of grazing intensity *G*_*p*_, assessed as the density of livestock (number per ha) and duration of pasture (days per year), amount of fertilizer application *F*_*p*_ (kg nitrogen per year and ha) and mowing intensity *M*_*p*_, defined as the number of cutting events per year. Each category *G*_*p*_, *F*_*p*_ and *M*_*p*_ was scaled by the mean of this variable over all sites from each of the three regions of the Biodiversity Exploratories ($\overline{G}$, $\overline{F}$ and $\overline{M}$, respectively). We used the mean of the *LUI* for the years 2006 to 2010, i.e. those preceding our experiment. There were eight subplots per plot, each measuring 1 × 1 m, which were planted with six phytometers of six different species, selected from a total pool of 130 species according to the four transplant addition scenarios, namely Sim, Dissim, Beals and Random. The six species planted in each subplot were specifically selected based on each plot’s species composition, and they therefore differed among plots. Species in the Sim and Dissim scenarios were selected that they would have respectively have the lowest and highest mean pairwise trait distance $\overline{d}$ to the extant species in each plot, with the selection based on eight functional traits (SLA, LDMC, height, leaf anatomy, leaf persistence, leaf distribution, vegetative reproduction, physical defense; see SI Table S1). These traits closely reflect the trait constellation of all resident and phytometer species in the study (SI Fig. S1). As we used only young plants in the experiment, and deliberately excluded plants at the germination stage, we focused on persistence traits and disregarded seed and reproduction traits. As such, traits were chosen that reflect competitive ability through their capacity to affect growth rates such as specific leaf area (SLA), leaf dry matter content (LDMC), height, leaf anatomy, leaf persistence and leaf distribution^51,66^. SLA and LDMC were somewhat correlated (r^2^ = 0.23) across all phytometer and resident species in the community, while both were uncorrelated with height (SI Fig. S1). Furthermore, we included traits that increased persistence after disturbance by providing the ability to colonize or re-colonize habitats through means of vegetative reproduction. We also included the trait of “physical defense mechanisms”, because it directly relates to land-use. Plants that have physical defense traits such as thorns or hooks are less likely to be grazed. Thus, the traits used represented independent axes of specialization. Trait distance calculations were based on all these eight traits using Gower’s distance. In the Beals scenario, species used had the highest probability of co-occurrence with the resident species in the German Vegetation Reference Database (GVRD)^25^, while in the Random scenario, the species were randomly selected from the species pool. We calculated both the mean pairwise trait distances among all resident species before the phytometer species were planted and between the six introduced species in the four scenarios (Beals, Dissim, Random and Sim) and all resident species.

In total, we planted 2592 individuals (3 Exploratories, 18 plots per Exploratory, 8 subplots per plot, 6 plant individuals per subplot). Detailed information of the experimental design and the scenarios is reported in a previous paper^24^. SI Table S1 shows the mean trait values of the phytometers planted under the four different scenarios. With the exception of SLA, all scenarios differed in their trait values. On average, of all species selected for the different scenarios, species in the Beals scenario had leaves that were to a higher degree hygromorphic and arranged in rosettes and reproduced more frequently vegetatively. In contrast, Sim species ranked highest in mesomorphic and evergreen leaf types and regular leaf distribution. Dissim species were tallest, more scleromorphic and they often had semi-rosettes. Finally, species chosen for the Random scenario had leaves with the highest LDMC, which were more summer-green (Table S1).

After planting in April 2012, the phytometers were monitored regularly for growth and survival in April, May, July, August and October 2012 and in May, June/July and September 2013. These eight monitoring events were numbered from zero to seven. At each date, we recorded height, aboveground plant projection area (calculated from two diameters using the ellipse formula), leaf length and number of leaves. At the last monitoring date in September 2013, aboveground biomass of all surviving plants was harvested, dried and weighed. Regressions of height and projection area on aboveground biomass at the time of harvest showed a high positive correlation of r = 0.47 and 0.78, respectively, indicating that our non-destructive variables were good proxies for aboveground biomass. Photographs were taken of fresh leaf samples for every individual and then analyzed using Image J (version 1.48e^67^, National Institutes of Health) to assess leaf area. Fresh leaf samples and aboveground biomass were dried for three days at 60 °C. Dry leaves and dry aboveground biomass were weighed and summed to calculate total aboveground biomass. SLA was calculated by dividing leaf area (m²) by dry leaf mass (kg). LDMC was calculated by dividing dry leaf mass (mg) by fresh leaf mass (g)^51^. As most species did not produce flowers or fruits in the presence of grazing and cutting, we could not analyze individual fitness but instead focused on relative growth rates (RGR) as a measure of performance. RGR was calculated according to formula (2)^68^, where M is any growth variable and t is the time span in weeks between the two monitoring dates 1 and 2.

2 $$RG{R}_{i}=\frac{\mathrm{ln}({M}_{2})-\,\mathrm{ln}({M}_{1})}{{t}_{2}-{t}_{1}}$$

We calculated RGR for three intervals, May to October 2012 (1–4), October 2012 to May 2013 (4–5), and May to September 2013 (5–7).

### CWM calculation

The community-weighted mean value (CWM) of SLA, LDMC and height was calculated according to formula (3):

3 $${\rm{CWM}}={\sum }_{i=1}^{s}{p}_{i}\ast {x}_{i}$$

where *p*_*i*_ is the relative cover of species *i* (*i* = *1, 2*, *…*, *s*) obtained from vegetation records on all subplots made in 2011, when the visual plant cover of every species was estimated as a percentage of the subplot area (1 m^2^). Total plant cover included the cover of transplants, obtained from the aboveground plant projection area calculated from two diameters using the ellipse formula and then transferred to percentage. As the six transplanted individuals contributed to the CWM trait value of the subplot, only the surviving transplants were included in the calculation. In addition, as the transplants were of different size on the various monitoring dates, CWMs differed among dates. The trait value (*x*_*i*_) of species *i* (*i* = *1, 2, …, s*) refer to species mean trait values measured in 2011, complemented from the databases LEDA^66^, BIOPOP^69^, BIOLFLOR^70^ and Rothmaler^71^. Species with missing trait values were excluded from CWM calculation.

### FD calculation

FD was calculated according to Rao´s defined quadratic entropy^72^ according to formula (4):

4 $$F{D}_{Q}={\sum }_{i=1}^{s}{\sum }_{j=i+1}^{S-1}{D}_{ij}\ast {p}_{i}\ast {p}_{j}$$

where *p*_*i*_ and *p*_*j*_ are relative cover and *D*_*ij*_ the trait distances between all species *i* and *j* in one subplot. The calculation employed the same traits as in CWM (SLA, LDMC and height) and the same multi-trait distance *D*_*ij*_ that was used for selecting the species for the Sim and Dissim scenario (see above). As in CWM, FD included the sizes of all survivors at the end of the three time intervals.

### Climate Data

Air temperature and relative air humidity were measured at 2 m above the ground, while soil temperature and moisture were measured at 10 cm below the ground. All climate data were collected at 10 minutes intervals using data loggers installed in the same plots and aggregated to monthly mean values (see Acknowledgements). The monthly means were averaged for the three time intervals in our study (vegetation period 2012, winter 2012/2013 and vegetation period 2013).

### Data Analysis

In a first step, to analyse the total amount of variation explained by our study design, we subjected all 15 response variables to a variance partitioning analysis (i.e. height, aboveground plant projection area, leaf length and number of leaves, for each of the three monitoring intervals as well as aboveground biomass, SLA and LDMC at the time of harvest). The exclusive and jointly explained variances by plot (nested in Exploratory: Schwäbische Alb, Hainich and Schorfheide), scenario and species identity were assessed using the varpart command in the vegan package in R^73^. We repeated the analysis including additional subplot-based trait measures to serve as a fourth predictor category (both CWM and FD of height, SLA and LDMC as well as FD of all traits).

Thereafter, regressions were calculated using mixed linear models in R (lmer, package lmerTest)^74^ using “Exploratory”, “plot” nested in “Exploratory”, “subplot” nested in “plot” and “species identity” as random factors. This random structure was used in all subsequent models.

In the second step, we calculated single fixed-predictor linear mixed effects models that related RGR of the 15 different response variables (height, plant projection area, leaf length, number of leaves, for each of the three monitoring intervals, and the variables at the time of harvest, i.e. aboveground biomass, SLA and LDMC) to eight predictor variables (CWM and FD of height, SLA and LDMC as well as to FD of all traits used in the scenario definition (multi-trait FD) and land-use intensity (LUI)). We identified the models with the highest absolute standardized estimates of the predictor and plotted standardized estimates for all 15 response variables.

In the third step, we constructed full multiple-predictor linear mixed effects models that related the RGR of the different response variables to land-use intensity (LUI), scenario (Beals, Dissim, Sim, Random), CWM (separately for SLA, LDMC and height) and FD (of the single traits SLA, LDMC and height and the multi-trait FD). Logger failure resulted in plots with missing values for one of the four climate variables (see SI Table S3), which precluded the inclusion of climate variables in the full models for all 54 plots. As we were particularly interested in how the different scenarios modified the phytometer responses, we also included all two-fold interactions with scenario. Aboveground biomass was log transformed to achieve normal distribution. The models were then improved by backward selection of predictor variables using the step command of R package lmerTest to eliminate insignificant effects^74^. The final models were then compared with models to which one of the four climate variables (air temperature, relative air humidity, soil temperature and soil moisture) was added as an additional predictor, using AIC. This required recalculating the models both with and without climate variables for the subsets for which climate data were available (SI Table S3). Models with climate variables were considered better than those without if ΔAIC was >1.

We used the method described by Nakagawa & Schielzeth to calculate the amount of variance explained by the mixed models^75^, both as marginal R^2^ (i.e. the variance explained by fixed factors), and as conditional R^2^ (i.e. the variance explained by both fixed and random factors). While all graphs were produced using unscaled variables, all predictor variables in the multiple regression models and those reported in the tables were scaled by mean and standard deviation, which allowed for the direct comparison of parameter estimates of effect sizes. All estimates refer to the Beals scenario (when scenario as a categorical predictor was included in the model Beals was coded as intercept in the model’s design matrix) and to the mean of all continuous variables in the final model. The parameter estimates of these final models were then used to calculate regression lines. Partial regressions were produced by calculating a linear mixed effects model without the target variable and then relating the residuals of that model to the target variable in an ordinary linear model. For all statistical analyses, we used the software R version 3.4.3^76^.

The datasets analysed during the current study are available from the corresponding author upon request; for trait values also see supporting information Table S2.

## Electronic supplementary material

Supplementary Information
