# Supplementary material for: Using co-occurrence information and trait composition to understand individual plant performance in grassland communities
Source: Sci Rep. 2018 Jun 13;8:9076. doi: 10.1038/s41598-018-27017-9 (PMC5998150; doi:10.1038/s41598-018-27017-9)
Supplement: Supplementary file 1 — Supplementary Information [file 41598_2018_27017_MOESM1_ESM.pdf]

Supporting information

Title: Using co-occurrence information and trait composition to understand individual plant performance in grassland communities

Authors: Eva Breitschwerdt[1], Ute Jandt[1, 2], *Helge Bruelheide[1, 2]

Addresses: 1: Martin Luther University Halle-Wittenberg, Institute of Biology / Geobotany and Botanical Garden, Am Kirchtor 1, 06108 Halle, Germany

2: German Centre for Integrative Biodiversity Research (iDiv) Halle-Jena-Leipzig, Deutscher Platz 5e, D-04103 Leipzig.

Email addresses: eva.breitschwerdt@botanik.uni-halle.de

ute.jandt@botanik.uni-halle.de

helge.bruelheide@botanik.uni-halle.de

Tables

Table S1: Differences in trait values between the different scenarios. F and p refer to an ANOVA. Est. = estimated trait value for the different scenarios. Small letters refer to statistically significant differences among scenarios according to a Tukey post-hoc test.

**Trait**	**Trait description**	**F**	**P**	**Est. Beals**	**Tukey**	**Est. Dissim**	**Tukey**	**Est. Random**	**Tukey**	**Est. Sim**	**Tukey**
Specific leaf area (SLA)	Leaf area per dry mass [m^2/kg]	1.76	0.15	20.36	a	20.45	a	20.44	a	21.06	a
Leaf dry matter content (LDMC)	Leaf dry mass per leaf fresh mass [mg/g]	42.98	0.00	248.18	b	223.52	d	264.06	a	235.10	c
Height	[m]	39.67	0.00	0.51	b	0.65	a	0.52	b	0.49	b
Leaf anatomy 01	leaf succulent	12.21	0.00	0.00	b	0.02	a	0.00	b	0.00	b
Leaf anatomy 02	scleromorphic	546.29	0.00	0.19	c	0.85	a	0.47	b	0.06	d
Leaf anatomy 03	mesomorphic	588.85	0.00	0.94	b	0.31	d	0.81	c	1.00	a
Leaf anatomy 04	hygromorphic	33.66	0.00	0.12	a	0.03	c	0.07	b	0.00	d
Leaf anatomy 05	helomorphic	10.10	0.00	0.05	b	0.02	c	0.04	cb	0.08	a
Leaf persistence 01	green before summer	16.82	0.00	0.00	b	0.04	a	0.03	a	0.00	b
Leaf persistence 02	green in summer	24.11	0.00	0.33	b	0.38	b	0.52	a	0.32	b
Leaf persistence 03	green in winter	50.56	0.00	0.00	c	0.10	a	0.03	b	0.00	c
Leaf persistence 04	evergreen	48.98	0.00	0.67	a	0.48	b	0.42	b	0.68	a
Leaf distribution 01	leaves distributed regularly	51.51	0.00	0.40	b	0.19	c	0.33	b	0.51	a
Leaf distribution 02	rosette	22.92	0.00	0.10	a	0.01	b	0.08	a	0.03	b
Leaf distribution 03	semi-rosette	64.55	0.00	0.50	c	0.80	a	0.59	b	0.46	c
Physical defense	Presence of hooks, spines, thorns, stinging hairs	148.05	0.00	0.01	b	0.22	a	0.02	b	0.00	b
Vegetative reproduction	Presence of stolons, rhizome, bulb, etc.	411.47	0.00	0.99	a	0.38	c	0.72	b	0.96	a

Table S2: Trait values of the 130 species transplanted into subplots and information on which of the four tested Scenarios the species were planted (B = Beals, D = Dissim, R = Random, S = Sim). The survival rate (Surv.) is the percentage of individuals alive at end of the experiment compared to the amount of individuals transplanted at the start. As an example of growth rate, RGR h4-5 is given. RGRh4-5 is the species mean across all individuals´ RGR height in the winter time interval (4-5) [cm cm $^{-1}$ week^{-1}]. NA (= not available) values for RGR height indicate that no individual of that species survived until the 5th monitoring interval. Trait values for each species include: L.A. = Leaf anatomy (categories 1 – 6); L.P. = Leaf persistence (categories 1 – 4); L.D. = Leaf distribution (categories 1 – 3); Veg. R. = Vegetative reproduction, and Phys. D. = Physical defense. For an explanation of trait categories, see SI Table S1.

Species	Freq.	Scen.	RGRh4-5	Surv.	SLA	LDMC	Height	L.A.1	L.A.2	L.A.3	L.A.4	L.A.5	L.A.6	L.P.1	L.P.2	L.P.3	L.P.4	L.D.1	L.D.2	L.D.3	Phys. D.	Veg. R.
Achillea millefolium	50	B,R	0.0033	0.78	11.86	274.50	0.45	0	1	1	0	0	0	0	0	0	1	0	0	1	0	1
Agrimonia eupatoria	6	B,R	-0.0143	0.50	13.15	383.12	0.38	0	0	1	0	0	0	0	1	0	0	0	0	1	0	1
Allium scorodoprasum	30	D,R	NA	0.00	11.57	255.09	0.56	0	0	1	0	0	0	1	0	0	0	0	0	1	0	1
Allium vineale	16	D,R	NA	1.00	17.61	137.75	0.30	0	0	1	0	0	0	1	0	0	0	1	0	0	0	1
Alopecurus pratensis	30	B,R	0.0142	0.17	16.82	355.17	0.70	0	0	1	0	0	0	0	1	0	0	1	0	0	0	1
Angelica sylvestris	6	R	NA	0.00	16.54	221.31	1.15	0	0	1	0	1	0	0	1	0	0	0	0	1	0	0
Anthoxanthum odoratum	56	B	0.0102	0.82	21.08	262.12	0.22	0	0	1	0	0	0	0	0	0	1	1	0	0	0	1
Anthyllis vulneraria	16	B,S	0.0223	0.19	13.58	227.61	0.32	0	0	1	0	0	0	0	0	0	1	0	0	1	0	0
Armeria maritima	6	R	-0.0122	0.00	15.89	247.83	0.27	0	1	0	0	0	0	0	0	0	1	0	1	0	0	0
Arrhenatherum elatius	46	B,R,S	0.0201	0.33	23.92	313.30	1.03	0	0	1	0	0	0	0	1	0	0	1	0	0	0	1
Artemisia campestris	8	R	NA	0.00	17.15	255.77	0.45	0	1	1	0	0	0	0	1	0	0	1	0	0	0	0
Artemisia vulgaris	12	D,R	0.0093	0.42	39.75	142.72	1.55	0	1	1	0	0	0	0	1	0	0	1	0	0	0	0
Asperula cynanchica	8	R	-0.0343	0.00	15.88	325.06	0.17	0	1	0	0	0	0	0	1	0	0	1	0	0	0	1
Astragalus glycyphyllos	12	S	-0.0135	0.33	32.51	184.27	1.00	0	0	1	0	0	0	0	1	0	0	1	0	0	0	1
Bellis perennis	52	B,R	0.0027	0.46	29.71	180.71	0.10	0	0	1	1	0	0	0	0	0	1	0	1	0	0	1
Betonica officinalis	14	R	0.0268	0.43	24.90	360.45	0.65	0	0	1	0	0	0	0	1	0	0	1	0	0	0	1

Brachypodium pinnatum	6	R,S	-0.0036	0.67	15.04	474.77	0.55	0	1	1	0	0	0	0	1	0	0	1	0	0	0	1
Briza media	60	B,D,R	0.0044	0.64	29.56	320.63	0.35	0	1	1	0	0	0	0	0	0	1	0	0	1	0	1
Bromus erectus	12	R	0.0044	0.67	13.04	342.88	0.60	0	1	1	0	0	0	0	0	0	1	0	0	1	0	1
Campanula patula	10	D,R	-0.0113	0.10	38.22	179.75	0.45	0	0	1	0	0	0	0	0	0	1	0	0	1	0	0
Campanula rotundifolia	4	B	0.0284	0.75	21.12	405.83	0.20	0	0	1	0	0	0	0	0	0	1	0	0	1	0	1
Carex flacca	8	R	-0.0105	0.25	10.71	514.33	0.40	0	0	1	0	0	0	0	0	0	1	0	0	1	0	1
Carlina vulgaris	26	D	-0.0233	0.12	12.34	312.85	0.37	0	1	0	0	0	0	0	0	0	1	0	0	1	1	0
Carum carvi	2	R	0.0185	0.50	19.14	248.72	0.55	0	0	1	0	0	0	0	1	0	0	0	0	1	0	0
Centaurea jacea	42	B,R	0.0179	0.60	10.60	255.55	0.82	0	1	1	0	0	0	0	1	0	0	0	0	1	0	1
Centaurea nigra	18	S	0.0261	0.50	14.81	300.81	0.45	0	0	1	0	0	0	0	1	0	0	0	0	1	0	1
Centaurea scabiosa	28	B,D,R	0.0056	0.50	30.07	140.55	0.85	0	1	0	0	0	0	0	1	0	0	0	0	1	0	1
Centaurea stoebe	72	D,R	-0.0016	0.22	18.28	198.45	0.85	0	1	0	0	0	0	0	0	0	1	0	0	1	0	0
Cerastium holosteoides	18	B,S	0.0133	0.36	23.35	250.92	0.27	0	0	1	0	0	0	0	0	0	1	1	0	0	0	1
Cichorium intybus	50	D,R	0.0216	0.40	22.73	174.86	0.62	0	1	0	0	0	0	0	1	0	0	0	0	1	0	1
Cirsium acaule	8	D,R	-0.0135	0.50	8.72	222.50	0.14	0	1	1	0	0	0	0	1	0	0	0	0	1	1	1
Cirsium arvense	4	B	-0.0354	0.25	12.03	203.27	0.90	0	1	1	0	0	0	0	1	0	0	1	0	0	1	1
Crepis biennis	6	R	0.0309	0.17	23.02	170.46	0.85	0	0	1	0	0	0	0	0	0	1	0	0	1	0	0
Crepis tectorum	22	D	0.0003	0.00	26.31	144.09	0.35	0	0	1	0	0	0	0	0	1	0	0	0	1	0	0
Cynosurus cristatus	8	B,R	0.0139	0.75	23.00	302.62	0.40	0	0	1	0	0	0	0	0	0	1	0	0	1	0	1
Daucus carota	4	R	-0.0055	0.50	15.11	262.57	0.55	0	0	1	0	0	0	0	0	0	1	0	0	1	0	0
Dianthus carthusianorum	36	D,R	0.0263	0.36	16.44	207.73	0.30	0	1	0	0	0	0	0	1	0	0	0	0	1	0	0
Dipsacus fullonum	98	D	0.0043	0.21	17.16	209.34	1.35	0	1	0	0	0	0	0	0	0	1	0	0	1	1	0
Echium vulgare	6	D	-0.0269	0.17	30.73	113.18	0.56	0	1	0	0	0	0	0	0	0	1	0	0	1	1	0
Erigeron acris	20	D,R	-0.0039	0.00	18.11	251.09	0.35	0	1	1	0	0	0	0	0	1	0	0	0	1	0	0
Euphorbia esula	42	R,S	NA	0.00	30.42	263.27	0.45	0	0	1	0	0	0	0	1	0	0	1	0	0	0	1
Festuca arundinacea	8	R	0.0037	0.50	20.27	382.86	1.20	0	1	1	0	0	0	0	1	0	0	1	0	0	0	1
Festuca guestfalica	2	R	-0.0130	1.00	9.62	501.39	0.30	0	1	0	0	0	0	0	0	0	1	0	0	1	0	1
Festuca nigrescens	6	R	NA	0.00	10.21	365.90	0.47	0	0	1	0	0	0	0	0	0	1	0	0	1	0	1
Festuca ovina	14	D,R	0.0060	0.71	14.01	301.00	0.26	0	1	0	0	0	0	0	0	0	1	0	0	1	0	1
Festuca pratensis	24	B,R	-0.0023	0.77	18.17	329.28	0.70	0	0	1	0	0	0	0	0	0	1	1	0	0	0	1

Festuca rubra	16	B	0.0085	0.79	10.21	365.90	0.51	0	0	1	0	0	0	0	0	0	1	0	0	1	0	1
Filipendula ulmaria	10	B,R	-0.0065	0.60	41.20	194.00	1.00	0	0	1	0	0	0	0	1	0	0	0	0	1	0	1
Galium mollugo	10	R,S	-0.0081	0.17	29.62	231.33	0.51	0	0	1	0	0	0	0	1	0	0	1	0	0	0	1
Galium pumilum	10	R	-0.0153	0.50	25.95	447.06	0.20	0	0	1	0	0	0	0	1	0	0	1	0	0	0	1
Geranium pratense	2	R	0.0251	1.00	14.47	266.40	0.40	0	0	1	1	0	0	0	1	0	0	0	0	1	0	1
Geranium pusillum	6	R	NA	0.00	25.48	205.14	0.22	0	0	1	0	0	0	0	0	1	0	0	0	1	0	0
Geranium pyrenaicum	2	R	0.0386	0.50	24.65	183.62	0.47	0	0	1	0	0	0	0	0	0	1	0	0	1	0	0
Geranium sylvaticum	2	R	-0.0171	1.00	18.31	247.53	0.40	0	0	1	1	0	0	0	1	0	0	0	0	1	0	1
Geum rivale	12	R	0.0109	0.67	25.29	221.98	0.50	0	0	1	0	0	0	0	1	0	0	0	0	1	0	1
Geum urbanum	12	R	-0.0059	0.83	14.15	312.29	0.75	0	0	1	1	0	0	0	0	0	1	0	0	1	0	1
Helianthemum nummularium	6	B,R	0.0112	0.33	15.19	252.92	0.15	0	1	0	0	0	0	0	0	0	1	1	0	0	0	1
Helichrysum arenarium	4	R	0.0000	0.00	18.17	265.53	0.20	0	1	0	0	0	0	0	1	0	0	0	0	1	0	1
Helictotrichon pubescens	8	R	0.0015	0.50	12.60	354.92	0.65	0	0	1	0	0	0	0	1	0	0	0	0	1	0	1
Hieracium pilosella	26	B,R,S	0.0170	0.50	14.18	295.25	0.17	0	0	1	0	0	0	0	0	0	1	0	1	0	0	1
Holcus lanatus	82	B,R,S	0.0155	0.83	28.19	267.25	0.65	0	0	1	0	0	0	0	0	0	1	1	0	0	0	1
Hypericum perforatum	14	B,R	-0.0003	0.64	18.23	366.68	0.56	0	1	1	0	0	0	0	1	0	0	1	0	0	0	1
Hypochaeris radicata	4	R	-0.0054	1.00	33.85	97.53	0.37	0	0	1	0	0	0	0	0	0	1	0	1	0	0	1
Jasione montana	6	R	0.0280	0.00	17.27	253.09	0.27	0	1	1	0	0	0	0	0	0	1	0	0	1	0	1
Knautia arvensis	14	B,R	0.0088	0.50	21.52	176.14	0.55	0	0	1	0	0	0	0	1	0	0	0	0	1	0	1
Koeleria pyramidata	4	R	-0.0065	1.00	13.71	325.52	0.65	0	1	1	0	0	0	0	1	0	0	0	0	1	0	1
Lathyrus pratensis	10	B	-0.0039	0.30	29.84	289.71	0.65	0	0	1	0	0	0	0	1	0	0	1	0	0	0	1
Leontodon autumnalis	16	B,R,S	-0.0005	0.50	26.07	171.37	0.30	0	0	1	0	0	0	0	0	0	1	0	1	0	0	1
Leontodon hispidus	12	B,R,S	0.0157	0.50	14.97	285.42	0.28	0	0	1	0	0	0	0	1	0	0	0	1	0	0	1
Leucanthemum vulgare	6	R	0.0476	0.50	22.67	138.10	0.50	0	1	1	0	0	0	0	0	0	1	0	0	1	0	1
Linum austriacum	18	R,S	0.0083	0.33	17.84	247.85	0.45	0	1	1	0	0	0	0	1	0	0	1	0	0	0	1
Lolium perenne	28	B,S	0.0084	0.44	18.86	251.79	0.35	0	0	1	0	0	0	0	0	0	1	0	0	1	0	1
Lotus corniculatus	72	B,R,S	-0.0041	0.56	20.03	252.42	0.22	0	0	1	0	0	0	0	1	0	0	1	0	0	0	1
Luzula campestris	30	B,R,S	0.0195	0.62	20.01	227.85	0.15	0	1	1	0	0	0	0	1	0	0	0	0	1	0	1
Malva alcea	4	R	-0.0175	0.25	23.12	214.01	0.82	0	0	1	0	0	0	0	1	0	0	0	0	1	0	0
Medicago falcata	4	S	0.0199	0.50	20.67	360.59	0.35	0	1	1	0	0	0	0	1	0	0	1	0	0	0	1

Medicago lupulina	12	R,S	0.0213	0.08	24.20	319.07	0.37	0	0	1	0	0	0	0	0	0	1	1	0	0	0	1
Medicago x varia	6	R	0.0046	0.83	24.56	239.90	0.55	0	0	1	0	0	0	0	0	0	1	1	0	0	0	1
Melilotus officinalis	14	R	-0.0353	0.00	17.33	218.30	0.65	0	1	1	0	0	0	0	1	0	0	1	0	0	0	0
Oenothera biennis	4	D	NA	0.00	15.93	238.30	1.00	0	1	1	0	0	0	0	0	0	1	0	0	1	0	1
Ononis spinosa	10	D,R	-0.0405	0.50	15.91	333.94	0.45	0	0	1	0	0	0	0	1	0	0	1	0	0	1	1
Origanum vulgare	10	R,S	-0.0116	0.60	13.24	358.58	0.40	0	1	1	0	0	0	0	1	0	0	1	0	0	0	1
Pastinaca sativa	28	D,R	0.0146	0.27	18.12	221.48	0.94	0	1	1	0	0	0	0	1	0	0	0	0	1	0	0
Petrorhagia prolifera	22	D	NA	0.00	17.96	268.57	0.30	0	1	0	0	0	0	0	0	1	0	0	0	1	0	0
Peucedanum officinale	4	R	NA	0.00	13.06	303.40	1.30	0	1	1	0	0	0	0	1	0	0	0	0	1	0	1
Phleum phleoides	10	D,R	0.0277	0.40	17.90	319.28	0.45	0	1	0	0	0	0	0	0	0	1	0	0	1	0	1
Picris hieracioides	20	R,S	0.0101	0.35	21.00	327.09	0.61	0	1	1	0	0	0	0	1	0	0	0	0	1	0	1
Pimpinella major	6	S	0.0174	0.67	17.93	233.00	0.60	0	0	1	0	0	0	0	1	0	0	0	0	1	0	0
Pimpinella saxifraga	8	B,R	-0.0169	0.25	12.00	364.91	0.32	0	1	1	0	0	0	0	1	0	0	0	0	1	0	0
Plantago lanceolata	12	B	0.0470	0.70	17.61	204.11	0.30	0	1	1	0	0	0	0	1	0	0	0	1	0	0	1
Plantago major	8	D,R	NA	0.00	15.38	201.99	0.21	0	1	1	0	0	0	0	1	0	0	0	1	0	0	1
Plantago media	6	R	0.0019	0.67	15.85	183.22	0.27	0	1	1	0	0	0	0	1	0	0	0	1	0	0	1
Poa angustifolia	4	R	-0.0075	0.25	13.37	427.66	0.60	0	1	1	0	0	0	0	0	0	1	0	0	1	0	1
Poa pratensis	4	B	-0.0011	0.75	12.04	374.79	0.55	0	0	1	0	0	0	0	0	0	1	1	0	0	0	1
Potentilla argentea	74	D,R	0.0018	0.39	19.10	281.85	0.34	0	1	0	0	0	0	0	1	0	0	1	0	0	0	0
Prunella vulgaris	56	B,R,S	0.0096	0.68	23.29	222.56	0.17	0	0	1	0	0	0	0	0	0	1	1	0	0	0	1
Pseudolysimachion spicatum	22	D,R	0.0167	0.50	41.11	128.24	0.27	0	1	0	0	0	0	0	0	0	1	1	0	0	0	1
Ranunculus acris	76	B,R,S	0.0131	0.61	14.50	256.95	0.75	0	0	1	0	0	0	0	1	0	0	0	0	1	0	1
Ranunculus bulbosus	2	R	0.0246	0.00	21.46	190.57	0.25	0	0	1	0	0	0	1	0	0	0	0	0	1	0	1
Ranunculus repens	40	B,R	0.0270	0.66	20.58	186.01	0.27	0	0	0	1	1	0	0	0	0	1	0	0	1	0	1
Rumex acetosa	126	B,R,S	0.0355	0.76	23.10	116.20	0.52	0	0	1	0	0	0	0	0	0	1	0	0	1	0	1
Rumex crispus	4	R,S	0.0399	0.75	15.36	199.38	0.90	0	0	1	0	1	0	0	0	0	1	0	0	1	0	0
Rumex obtusifolius	16	D,R	0.0161	0.63	23.98	160.04	0.85	0	0	1	1	0	0	0	0	0	1	0	0	1	0	0
Rumex thyrsiflorus	12	R,S	0.0285	0.67	18.36	208.54	0.75	0	0	1	0	0	0	0	0	0	1	0	0	1	0	0
Salvia pratensis	26	D,R	0.0190	0.35	20.38	186.37	0.45	0	1	1	0	0	0	0	1	0	0	0	0	1	0	1
Sanguisorba minor	8	B,R	-0.0008	0.25	13.63	307.29	0.32	0	1	1	0	0	0	0	0	0	1	0	0	1	0	0

Saponaria officinalis	6	R	0.0173	0.67	20.09	363.46	0.55	0	0	1	0	0	0	0	1	0	0	1	0	0	0	1
Scabiosa columbaria	18	B,R	-0.0073	0.33	16.00	217.36	0.45	0	1	1	0	0	0	0	0	0	1	0	0	1	0	0
Scirpus sylvaticus	52	S	-0.0068	0.25	21.60	267.20	0.65	0	0	1	0	1	0	0	0	0	1	0	0	1	0	1
Sedum maximum	12	D	0.0475	0.17	17.74	172.98	0.55	1	0	0	0	0	0	0	1	0	0	1	0	0	0	1
Senecio jacobaea	56	R,S	-0.0008	0.64	13.40	258.00	0.51	0	0	1	0	0	0	0	0	0	1	0	0	1	0	1
Silaum silaus	2	S	0.0017	1.00	13.93	293.66	0.65	0	1	1	0	0	0	0	1	0	0	0	0	1	0	1
Silene dioica	4	R	0.0117	0.50	37.11	133.14	0.60	0	0	1	0	0	0	0	0	0	1	0	0	1	0	0
Silene flos-cuculi	64	B,R,S	0.0358	0.63	20.07	186.34	0.55	0	0	1	0	0	0	0	0	0	1	0	0	1	0	1
Silene latifolia	66	R,S	0.0191	0.39	19.93	170.90	0.64	0	0	1	0	0	0	0	0	0	1	1	0	0	0	1
Silene otites	30	D,R	0.0050	0.30	15.41	263.07	0.40	0	1	0	0	0	0	0	0	0	1	0	0	1	0	1
Silene vulgaris	4	R	-0.0114	0.50	22.70	202.72	0.35	0	1	1	0	0	0	0	0	0	1	1	0	0	0	0
Symphytum officinale	6	R	0.0171	0.33	16.73	117.82	0.75	0	0	0	1	1	0	0	1	0	0	0	0	1	1	0
Tanacetum vulgare	2	R	0.0163	0.00	17.25	262.16	0.90	0	1	0	0	0	0	0	1	0	0	1	0	0	0	1
Thymus pulegioides	12	R,S	-0.0066	0.25	17.05	355.82	0.25	0	1	0	0	0	0	0	0	0	1	1	0	0	0	1
Torilis japonica	10	R	0.0016	0.10	22.45	399.73	0.75	0	0	1	0	0	0	0	0	0	1	0	0	1	0	0
Tragopogon pratensis	6	R	0.0145	0.33	34.42	203.53	0.38	0	0	1	0	0	0	0	1	0	0	0	0	1	0	0
Trifolium dubium	10	D,R	NA	0.00	35.00	236.22	0.17	0	0	1	0	0	0	0	0	1	0	1	0	0	0	0
Trifolium montanum	2	R	NA	0.00	21.65	281.33	0.27	0	1	1	0	0	0	0	1	0	0	0	0	1	0	0
Trisetum flavescens	50	B,R,S	0.0092	0.58	25.05	312.49	0.55	0	0	1	0	0	0	0	0	0	1	1	0	0	0	1
Valeriana officinalis	18	D,R	0.0098	0.44	30.56	122.96	1.15	0	0	0	1	1	0	0	1	0	0	0	0	1	0	1
Veronica teucrium	4	R	-0.0229	0.50	20.34	238.47	0.60	0	1	1	0	0	0	0	1	0	0	1	0	0	0	1
Vicia cracca	18	B,R	0.0051	0.11	20.81	298.67	0.75	0	0	1	0	0	0	0	1	0	0	1	0	0	0	1
Vicia sepium	4	S	0.0360	1.00	23.41	236.04	0.45	0	0	1	0	0	0	0	0	0	1	1	0	0	0	1
Vicia villosa	4	D,R	0.0237	0.00	42.45	234.87	0.35	0	0	1	0	0	0	0	0	1	0	1	0	0	0	0

Table S3: Number of included plots for calculating the mean of climatic variables.

	Air temperature	Soil temperature	Relative air humidity	Soil moisture
Veg. Period 2012	n = 48	n = 41	n = 46	n = 49
Winter 2012/2013	n = 51	n = 50	n = 51	n = 53
Veg. Period 2013	n = 52	n = 50	n = 52	n = 52

Table S4: Variance partitioning of RGR for all variables at all time intervals (1 - 4 = vegetation period 2012; 4 - 5 = winter 2012/2013; 5 - 7 = vegetation period 2013), and aboveground biomass, LDMC and SLA at the final harvest in September 2013), exclusively explained by plot (a), scenario (b), species (c) traits (d), and jointly by two (e, f, g, h, i, j) or three of these factors (k, l, m, n) or all of them (o), as well as residual variance (p). For an illustration of variance components a to p see Fig. 1. All components add up to 1. p. proj. area = plant projection area. Traits included Multi-trait FD, SLA FD/CWM, LDMC FD/CWM and Height FD /CWM.

	**a**	**b**	**c**	**d**	**e**	**f**	**g**	**h**	**i**	**j**	**k**	**l**	**m**	**n**	**o**	**p**	**Plot**	**Scenario**	**Species**	**Trait**
RGR height 1 - 4 ~ Multi-trait FD	0.126	0	0.375	0	0	0.042	0	0.001	0	0.001	0	0.003	0.008	0.004	0	0.452	0.123	0.051	0.422	0.012
RGR height 1 - 4 ~ SLA FD	0.127	0	0.375	0	0	0.049	0	0	0	0.001	0	0.003	0.001	0	0	0.452	0.123	0.051	0.422	0.001
RGR height 1 - 4 ~ LDMC FD	0.128	0	0.376	0	0	0.048	0	0	0	0	0	0.002	0.001	0.002	0	0.452	0.123	0.051	0.422	0.003
RGR height 1 - 4 ~ Height FD	0.109	0	0.376	0	0	0.049	0	0.017	0	0	0	0	0.001	0	0.005	0.452	0.123	0.051	0.422	0.018
RGR height 1 - 4 ~ SLA CWM	0.127	0	0.375	0	0	0.049	0	0	0	0	0	0.002	0	0.001	0	0.452	0.123	0.051	0.422	0.003
RGR height 1 - 4 ~ LDMC CWM	0.127	0	0.376	0	0	0.05	0	0	0	0	0	0.002	0	0	0	0.452	0.123	0.051	0.422	0
RGR height 1 - 4 ~ Height CWM	0.125	0	0.376	0	0	0.05	0	0.002	0	0	0	0.001	0	0	0	0.452	0.123	0.051	0.422	0
RGR p. proj. area 1 - 4 ~ Multi-trait FD	0.126	0.001	0.199	0.005	0	0	0.01	0	0	0.004	0	0.001	0.001	0	0	0.665	0.127	0	0.209	0
RGR p. proj. area 1 - 4 ~ SLA FD	0.122	0	0.2	0.001	0	0	0.009	0	0	0.003	0	0	0	0	0	0.669	0.127	0	0.209	0
RGR p. proj. area 1 - 4 ~ LDMC FD	0.123	0.001	0.2	0.002	0	0	0.008	0	0	0.003	0	0	0	0	0	0.668	0.127	0	0.209	0
RGR p. proj. area 1 - 4 ~ Height FD	0.098	0	0.203	0	0	0	0.012	0.024	0	0	0	0	0	0	0.001	0.67	0.127	0	0.209	0.018
RGR p. proj. area 1 - 4 ~ SLA CWM	0.122	0	0.203	0	0	0	0.001	0	0	0	0	0	0	0.005	0	0.67	0.127	0	0.209	0.004
RGR p. proj. area 1 - 4 ~ LDMC CWM	0.122	0.001	0.202	0.002	0	0	0	0	0	0.001	0	0	0	0.006	0	0.667	0.127	0	0.209	0.008
RGR p. proj. area 1 - 4 ~ Height CWM	0.109	0	0.202	0	0	0	0.008	0.012	0	0.001	0	0	0	0	0	0.669	0.127	0	0.209	0.013
RGR leaf length 1 - 4 ~ Multi-trait FD	0.129	0.004	0.205	0.002	0	0.002	0.003	0	0	0.001	0.001	0.004	0.002	0	0	0.656	0.13	0.008	0.213	0
RGR leaf length 1 - 4 ~ SLA FD	0.126	0.003	0.205	0	0	0.004	0.003	0	0	0	0	0.002	0	0	0	0.658	0.13	0.008	0.213	0
RGR leaf length 1 - 4 ~ LDMC FD	0.126	0.004	0.205	0	0	0.004	0.001	0.001	0	0	0	0.002	0	0.001	0	0.658	0.13	0.008	0.213	0.003
RGR leaf length 1 - 4 ~ Height FD	0.105	0.003	0.206	0.001	0	0.003	0.004	0.022	0	0	0	0	0	0	0.002	0.657	0.13	0.008	0.213	0.021
RGR leaf length 1 - 4 ~ SLA CWM	0.129	0.003	0.206	0.002	0	0.003	0.002	0	0	0	0	0.002	0	0	0	0.656	0.13	0.008	0.213	0
RGR leaf length 1 - 4 ~ LDMC CWM	0.126	0.003	0.206	0	0	0.004	0	0.001	0	0	0	0.002	0	0.002	0	0.658	0.13	0.008	0.213	0.003

RGR leaf length 1 - 4 ~ Height CWM	0.114	0.003	0.206	0	0	0.004	0.001	0.012	0	0	0	0	0	0.001	0.001	0.658	0.13	0.008	0.213	0.015
RGR leaf number 1 - 4 ~ Multi-trait FD	0.043	0.002	0.202	0	0	0.009	0.014	0	0	0	0	0	0	0	0	0.731	0.057	0.01	0.224	0
RGR leaf number 1 - 4 ~ SLA FD	0.044	0.002	0.202	0	0	0.009	0.014	0	0	0	0	0	0	0	0	0.731	0.057	0.01	0.224	0
RGR leaf number 1 - 4 ~ LDMC FD	0.044	0.002	0.202	0	0	0.009	0.014	0	0	0	0	0	0	0	0	0.731	0.057	0.01	0.224	0
RGR leaf number 1 - 4 ~ Height FD	0.044	0.002	0.202	0	0	0.009	0.014	0	0	0	0	0	0	0	0	0.731	0.057	0.01	0.224	0
RGR leaf number 1 - 4 ~ SLA CWM	0.044	0.001	0.203	0.002	0	0.008	0.015	0	0	0	0	0	0	0	0	0.729	0.057	0.01	0.224	0
RGR leaf number 1 - 4 ~ LDMC CWM	0.043	0.001	0.202	0	0	0.009	0.015	0.001	0	0	0	0	0	0	0	0.73	0.057	0.01	0.224	0
RGR leaf number 1 - 4 ~ Height CWM	0.043	0.002	0.202	0	0	0.009	0.015	0	0	0	0	0	0	0	0	0.731	0.057	0.01	0.224	0
RGR height 4 - 5 ~ Multi-trait FD	0.351	0.002	0.145	0	0.001	0.033	0	0.006	0	0	0	0	0	0	0	0.479	0.342	0.022	0.163	0.003
RGR height 4 - 5 ~ SLA FD	0.353	0.001	0.146	0	0.001	0.033	0	0.003	0	0	0	0	0	0	0	0.479	0.342	0.022	0.163	0.003
RGR height 4 - 5 ~ LDMC FD	0.348	0.001	0.145	0	0	0.033	0	0.008	0	0.001	0.001	0	0	0	0	0.479	0.342	0.022	0.163	0.007
RGR height 4 - 5 ~ Height FD	0.334	0.001	0.146	0.001	0.001	0.033	0	0.022	0	0	0	0	0	0.003	0.002	0.477	0.342	0.022	0.163	0.027
RGR height 4 - 5 ~ SLA CWM	0.359	0.001	0.146	0.002	0.001	0.033	0	0	0	0	0	0	0	0	0	0.477	0.342	0.022	0.163	0
RGR height 4 - 5 ~ LDMC CWM	0.352	0.002	0.146	0	0.001	0.033	0	0.005	0	0	0	0	0	0	0.001	0.478	0.342	0.022	0.163	0.004
RGR height 4 - 5 ~ Height CWM	0.352	0.001	0.146	0	0.001	0.032	0	0.004	0	0	0	0	0	0.001	0	0.479	0.342	0.022	0.163	0.006
RGR p. proj. area 4 - 5 ~ Multi-trait FD	0.204	0	0.135	0.001	0.001	0.049	0.012	0.001	0	0	0	0	0	0	0	0.61	0.206	0.038	0.185	0
RGR p. proj. area 4 - 5 ~ SLA FD	0.206	0	0.136	0.001	0	0.049	0.01	0	0	0	0	0	0	0.002	0	0.61	0.206	0.038	0.185	0
RGR p. proj. area 4 - 5 ~ LDMC FD	0.205	0	0.135	0.003	0	0.05	0.011	0	0	0	0	0	0	0.001	0	0.608	0.206	0.038	0.185	0.002
RGR p. proj. area 4 - 5 ~ Height FD	0.204	0	0.135	0.003	0.001	0.051	0.01	0.001	0	0	0	0	0	0.003	0.003	0.607	0.206	0.038	0.185	0.007
RGR p. proj. area 4 - 5 ~ SLA CWM	0.203	0	0.136	0.004	0	0.049	0.014	0.001	0	0	0	0	0	0	0	0.607	0.206	0.038	0.185	0.001
RGR p. proj. area 4 - 5 ~ LDMC CWM	0.199	0	0.135	0	0.001	0.049	0.015	0.005	0	0	0	0	0	0	0.001	0.611	0.206	0.038	0.185	0.002
RGR p. proj. area 4 - 5 ~ Height CWM	0.203	0	0.135	0	0.001	0.049	0.011	0.001	0	0	0	0	0	0.002	0.001	0.611	0.206	0.038	0.185	0.003
RGR leaf length 4 - 5 ~ Multi-trait FD	0.27	0	0.106	0	0	0.025	0.032	0.005	0	0	0	0	0	0.006	0	0.565	0.305	0.016	0.161	0.009
RGR leaf length 4 - 5 ~ SLA FD	0.273	0	0.106	0	0	0.025	0.035	0.003	0	0	0	0	0	0.003	0	0.566	0.305	0.016	0.161	0.005
RGR leaf length 4 - 5 ~ LDMC FD	0.266	0	0.105	0	0	0.025	0.025	0.009	0	0	0	0	0	0.012	0	0.565	0.305	0.016	0.161	0.022
RGR leaf length 4 - 5 ~ Height FD	0.265	0	0.106	0.001	0	0.026	0.033	0.011	0	0	0	0	0	0.004	0.003	0.564	0.305	0.016	0.161	0.018
RGR leaf length 4 - 5 ~ SLA CWM	0.278	0	0.107	0.002	0	0.025	0.035	0	0	0	0	0	0	0.003	0	0.563	0.305	0.016	0.161	0.001
RGR leaf length 4 - 5 ~ LDMC CWM	0.274	0	0.106	0	0	0.025	0.039	0.002	0	0	0	0	0	0	0	0.565	0.305	0.016	0.161	0
RGR leaf length 4 - 5 ~ Height CWM	0.272	0	0.106	0	0	0.025	0.031	0.004	0	0	0	0	0	0.006	0.002	0.566	0.305	0.016	0.161	0.011
RGR leaf number 4 - 5 ~ Multi-trait FD	0.091	0	0.202	0	0.001	0.019	0.049	0.001	0	0	0	0	0	0.004	0.001	0.642	0.139	0.012	0.266	0.005

RGR leaf number 4 - 5 ~ SLA FD	0.087	0	0.201	0	0.001	0.019	0.046	0.005	0	0	0	0	0	0.007	0	0.642	0.139	0.012	0.266	0.011
RGR leaf number 4 - 5 ~ LDMC FD	0.089	0	0.201	0	0.001	0.019	0.048	0.003	0	0	0	0	0	0.005	0.001	0.642	0.139	0.012	0.266	0.008
RGR leaf number 4 - 5 ~ Height FD	0.093	0	0.202	0.002	0.001	0.02	0.053	0	0	0	0	0	0	0	0	0.64	0.139	0.012	0.266	0.001
RGR leaf number 4 - 5 ~ SLA CWM	0.09	0	0.202	0	0.001	0.019	0.054	0.002	0	0	0	0	0	0	0	0.642	0.139	0.012	0.266	0.001
RGR leaf number 4 - 5 ~ LDMC CWM	0.089	0	0.202	0	0.001	0.019	0.053	0.003	0	0	0	0	0	0	0	0.642	0.139	0.012	0.266	0.003
RGR leaf number 4 - 5 ~ Height CWM	0.091	0	0.202	0	0.001	0.019	0.051	0.001	0	0	0	0	0	0.002	0	0.642	0.139	0.012	0.266	0.001
RGR height 5 - 7 ~ Multi-trait FD	0.375	0.002	0.16	0	0.004	0.016	0.017	0.016	0	0	0	0	0	0	0	0.423	0.4	0.012	0.182	0.01
RGR height 5 - 7 ~ SLA FD	0.379	0.002	0.159	0	0.004	0.016	0.01	0.011	0	0.001	0	0	0.001	0.005	0	0.422	0.4	0.012	0.182	0.017
RGR height 5 - 7 ~ LDMC FD	0.36	0.002	0.16	0	0.002	0.016	0.014	0.031	0	0	0.002	0	0	0.001	0	0.422	0.4	0.012	0.182	0.029
RGR height 5 - 7 ~ Height FD	0.388	0.002	0.16	0	0.004	0.016	0.013	0.003	0	0	0	0	0	0.003	0	0.422	0.4	0.012	0.182	0.005
RGR height 5 - 7 ~ SLA CWM	0.387	0.002	0.16	0	0.004	0.016	0.012	0.003	0	0	0	0	0	0.003	0	0.422	0.4	0.012	0.182	0.005
RGR height 5 - 7 ~ LDMC CWM	0.391	0.002	0.16	0	0.004	0.016	0.015	0	0	0	0	0	0	0	0.001	0.423	0.4	0.012	0.182	0.001
RGR height 5 - 7 ~ Height CWM	0.39	0.002	0.159	0	0.003	0.017	0.014	0.001	0	0	0	0	0	0.002	0.001	0.422	0.4	0.012	0.182	0.004
RGR p. proj. area 5 - 7 ~ Multi-trait FD	0.181	0	0.159	0.001	0.001	0.003	0	0.007	0	0	0	0	0	0.002	0.001	0.656	0.183	0	0.155	0.01
RGR p. proj. area 5 - 7 ~ SLA FD	0.177	0	0.157	0	0.001	0.002	0	0.011	0	0.002	0	0	0	0.007	0	0.657	0.183	0	0.155	0.019
RGR p. proj. area 5 - 7 ~ LDMC FD	0.176	0	0.159	0	0	0.002	0	0.012	0	0	0.001	0	0	0.004	0	0.658	0.183	0	0.155	0.015
RGR p. proj. area 5 - 7 ~ Height FD	0.187	0	0.159	0	0.001	0.002	0	0.001	0	0	0	0	0	0.001	0	0.658	0.183	0	0.155	0.001
RGR p. proj. area 5 - 7 ~ SLA CWM	0.183	0	0.159	0	0.001	0.002	0	0.005	0	0	0	0	0	0.006	0	0.658	0.183	0	0.155	0.01
RGR p. proj. area 5 - 7 ~ LDMC CWM	0.189	0	0.158	0.001	0.001	0.002	0	0	0	0.001	0	0	0	0	0	0.657	0.183	0	0.155	0
RGR p. proj. area 5 - 7 ~ Height CWM	0.184	0	0.158	0	0.001	0.002	0	0.004	0	0.001	0	0	0	0.003	0	0.658	0.183	0	0.155	0.007
RGR leaf length 5 - 7 ~ Multi-trait FD	0.312	0	0.097	0.004	0	0.001	0.043	0	0	0	0	0.005	0.001	0.008	0	0.533	0.365	0.003	0.152	0.01
RGR leaf length 5 - 7 ~ SLA FD	0.289	0	0.095	0.004	0	0.002	0.038	0.023	0	0.002	0	0.001	0	0.013	0.001	0.533	0.365	0.003	0.152	0.043
RGR leaf length 5 - 7 ~ LDMC FD	0.295	0	0.098	0.001	0	0.002	0.029	0.017	0	0	0	0.004	0	0.022	0	0.536	0.365	0.003	0.152	0.038
RGR leaf length 5 - 7 ~ Height FD	0.311	0	0.097	0	0	0.002	0.049	0.001	0	0	0	0.002	0	0.003	0	0.538	0.365	0.003	0.152	0.003
RGR leaf length 5 - 7 ~ SLA CWM	0.307	0	0.097	0	0	0.003	0.041	0.005	0	0	0	0.003	0	0.01	0	0.537	0.365	0.003	0.152	0.014
RGR leaf length 5 - 7 ~ LDMC CWM	0.313	0	0.096	0	0	0.003	0.051	0	0	0.001	0	0.002	0	0.001	0	0.537	0.365	0.003	0.152	0
RGR leaf length 5 - 7 ~ Height CWM	0.32	0	0.094	0.007	0	0.002	0.052	0	0	0.003	0	0.002	0	0	0	0.53	0.365	0.003	0.152	0.002
RGR leaf number 5 - 7 ~ Multi-trait FD	0.079	0	0.193	0	0	0.007	0.014	0.004	0	0	0	0	0	0	0	0.711	0.092	0.002	0.209	0.001
RGR leaf number 5 - 7 ~ SLA FD	0.08	0	0.188	0	0	0.007	0.008	0.003	0	0.005	0	0	0	0.004	0	0.711	0.092	0.002	0.209	0.011
RGR leaf number 5 - 7 ~ LDMC FD	0.083	0	0.193	0	0	0.007	0.011	0	0	0	0	0	0	0.001	0	0.711	0.092	0.002	0.209	0

RGR leaf number 5 - 7 ~ Height FD	0.083	0	0.193	0	0	0.007	0.012	0	0	0	0	0	0	0	0	0.711	0.092	0.002	0.209	0
RGR leaf number 5 - 7 ~ SLA CWM	0.08	0	0.189	0	0	0.006	0.013	0.003	0	0.003	0	0	0.001	0	0	0.711	0.092	0.002	0.209	0.006
RGR leaf number 5 - 7 ~ LDMC CWM	0.083	0	0.191	0	0	0.007	0.014	0	0	0.002	0	0	0	0	0	0.711	0.092	0.002	0.209	0
RGR leaf number 5 - 7 ~ Height CWM	0.079	0	0.193	0	0	0.007	0.013	0.003	0	0	0	0	0	0	0	0.711	0.092	0.002	0.209	0.002
Biomass ~ Multi-trait FD	0.101	0	0.222	0.001	0	0.002	0.053	0.02	0	0	0	0	0	0.008	0.001	0.593	0.184	0.002	0.285	0.03
Biomass ~ SLA FD	0.12	0	0.22	0	0	0.001	0.057	0.001	0	0.002	0	0	0	0.004	0.001	0.595	0.184	0.002	0.285	0.008
Biomass ~ LDMC FD	0.098	0	0.222	0	0	0.001	0.052	0.023	0	0	0.001	0.001	0	0.009	0	0.595	0.184	0.002	0.285	0.032
Biomass ~ Height FD	0.121	0	0.223	0	0	0.001	0.058	0	0	0	0	0	0	0.003	0	0.594	0.184	0.002	0.285	0.003
Biomass ~ SLA CWM	0.091	0	0.222	0	0.001	0.001	0.026	0.03	0	0	0	0	0	0.035	0.001	0.595	0.184	0.002	0.285	0.065
Biomass ~ LDMC CWM	0.11	0	0.22	0	0.001	0.001	0.031	0.011	0	0.002	0	0	0	0.029	0.001	0.594	0.184	0.002	0.285	0.043
Biomass ~ Height CWM	0.102	0	0.222	0.002	0	0.001	0.053	0.02	0	0	0	0.002	0	0.008	0	0.592	0.184	0.002	0.285	0.029
LDMC ~ Multi-trait FD	0.134	0.002	0.298	0	0	0.016	0.043	0	0	0.001	0	0.014	0	0	0.004	0.492	0.192	0.032	0.373	0.004
LDMC ~ SLA FD	0.13	0.002	0.298	0.001	0	0.015	0.04	0.004	0	0.001	0	0.017	0	0.003	0.001	0.491	0.192	0.032	0.373	0.008
LDMC ~ LDMC FD	0.128	0.002	0.3	0	0	0.014	0.035	0.006	0	0	0	0.015	0	0.008	0.002	0.492	0.192	0.032	0.373	0.015
LDMC ~ Height FD	0.132	0.002	0.299	0	0	0.014	0.045	0.002	0	0	0	0.018	0.001	0	0	0.492	0.192	0.032	0.373	0
LDMC ~ SLA CWM	0.131	0.002	0.299	0	0	0.014	0.034	0.003	0	0	0	0.016	0	0.009	0.001	0.493	0.192	0.032	0.373	0.011
LDMC ~ LDMC CWM	0.13	0.002	0.298	0	0	0.013	0.042	0.004	0	0.001	0	0.015	0.001	0.001	0.002	0.492	0.192	0.032	0.373	0.008
LDMC ~ Height CWM	0.134	0.002	0.299	0	0	0.014	0.041	0	0	0	0	0.018	0	0.002	0	0.493	0.192	0.032	0.373	0
SLA ~ Multi-trait FD	0.19	0	0.224	0	0	0.023	0.008	0.001	0	0	0	0.023	0.001	0.007	0.003	0.522	0.231	0.048	0.289	0.012
SLA ~ SLA FD	0.181	0	0.224	0	0	0.024	0.018	0.01	0	0	0	0.027	0	0	0	0.522	0.231	0.048	0.289	0.005
SLA ~ LDMC FD	0.186	0	0.225	0	0	0.024	0.005	0.006	0	0	0	0.024	0	0.01	0.002	0.522	0.231	0.048	0.289	0.017
SLA ~ Height FD	0.193	0	0.225	0.003	0	0.024	0.015	0	0	0	0	0.026	0.001	0	0	0.519	0.231	0.048	0.289	0
SLA ~ SLA CWM	0.187	0	0.224	0	0	0.024	0	0.004	0	0	0	0.025	0	0.021	0.001	0.522	0.231	0.048	0.289	0.025
SLA ~ LDMC CWM	0.187	0	0.224	0	0	0.024	0.005	0.004	0	0	0	0.02	0	0.01	0.006	0.522	0.231	0.048	0.289	0.019
SLA ~ Height CWM	0.198	0	0.224	0.006	0	0.026	0.012	0	0	0	0	0.026	0	0.003	0	0.516	0.231	0.048	0.289	0.001

35

Table S5: Final models and their relative proportion of variances explained by the random factors Exploratory, plot (nested in Exploratory), subplot (nested in plot) and species identity (crossed with the other random factors) as well as residual variance. p. proj. area = plant projection area.

**Responses**	**Predictors**	**Variance of random factors %**				
**Veg. period 2012**		**Explo**	**Plot**	**Subplot**	**Species**	**Residuals**
RGR height	Multi-trait FD	0.066	0.098	0.031	0.340	0.466
RGR p. proj. area	Multi-trait FD	0.011	0.113	0.036	0.254	0.586
RGR leaf length	Height FD + Scen	0.028	0.090	0.019	0.241	0.622
RGR leaf number	SLA FD * Scen	0.006	0.047	0.009	0.257	0.681
**Winter 2012/2013**						
RGR height	LUI + Height FD * Scen + Height CWM * Scen	0.285	0.163	0.039	0.120	0.393
RGR p. proj. area	LUI + Height FD * Scen + SLA CWM + LDMC CWM + Height CWM	0.123	0.124	0.021	0.191	0.541
RGR leaf length	LUI + Height FD * Scen + Height CWM * Scen	0.245	0.126	0.042	0.107	0.481
RGR leaf number	LUI + Height FD + SLA CWM * Scen	0.029	0.077	0.000	0.326	0.567
**Veg. period 2013**						
RGR height	LUI	0.375	0.135	0.019	0.150	0.322
RGR p. proj. area	SLA FD	0.127	0.102	0.005	0.160	0.605
RGR leaf length	SLA FD + Height CWM	0.331	0.130	0.002	0.093	0.443
RGR leaf number	Multi-trait FD + SLA FD	0.017	0.058	0.022	0.251	0.652
**Harvest 2013**						
Biomass	LUI + Multi-trait FD * Scen + LDMC FD * Scen + SLA CWM * Scen	0.000	0.097	0.001	0.286	0.617
SLA	Height CWM + LDMC CWM * Scen	0.102	0.173	0.001	0.209	0.514
LDMC	SLA FD + LDMC FD + SLA CWM * Scen + LDMC CWM * Scen	0.000	0.148	0.009	0.383	0.460

Table S6: Final models that were improved with respect to AIC by adding climate variables: soil moisture (SM) and relative air humidity (rH). These models were only calculated for plots, for which climate data were available (for number of plots see SI Table S3). In all cases, the predictors remained significant after the climate variable had been added.

Responses	Predictors	Added c. variable	AIC without c. variable	AIC with c. variable	Estimate c. variable	P-value c. variable
**Winter 2012/2013**						
RGR height	LUI + Height FD * Scen + Height CWM * Scen	SM	3340.5	3334.6	-0.0206	0.0050
RGR p. proj. area	LUI + Height FD * Scen + SLA CWM + LDMC CWM + Height CWM	SM	3724.4	3712.4	-0.0232	0.0002
RGR leaf length	LUI + Height FD * Scen + Height CWM * Scen	SM	3551.3	3543	-0.0210	0.0014
RGR leaf number	LUI + Height FD + SLA CWM * Scen	SM	3781.1	3765.3	-0.0208	0.0000
**Veg. period 2013**						
RGR height	LUI	SM	2529.3	2525.7	0.0157	0.0183
RGR p. proj. Area	SLA FD	SM	2943.6	2941.5	0.0123	0.0431
RGR leaf length	SLA FD + Height CWM	SM	2614.6	2609.4	0.0177	0.0074
**Harvest 2013**						
SLA	Height CWM + LDMC CWM * Scen	SM	2634.3	2620	0.0274	0.0001
LDMC	SLA FD + LDMC FD + SLA CWM * Scen + LDMC CWM * Scen	rH	2616	2613.1	0.0284	0.0296

Figures

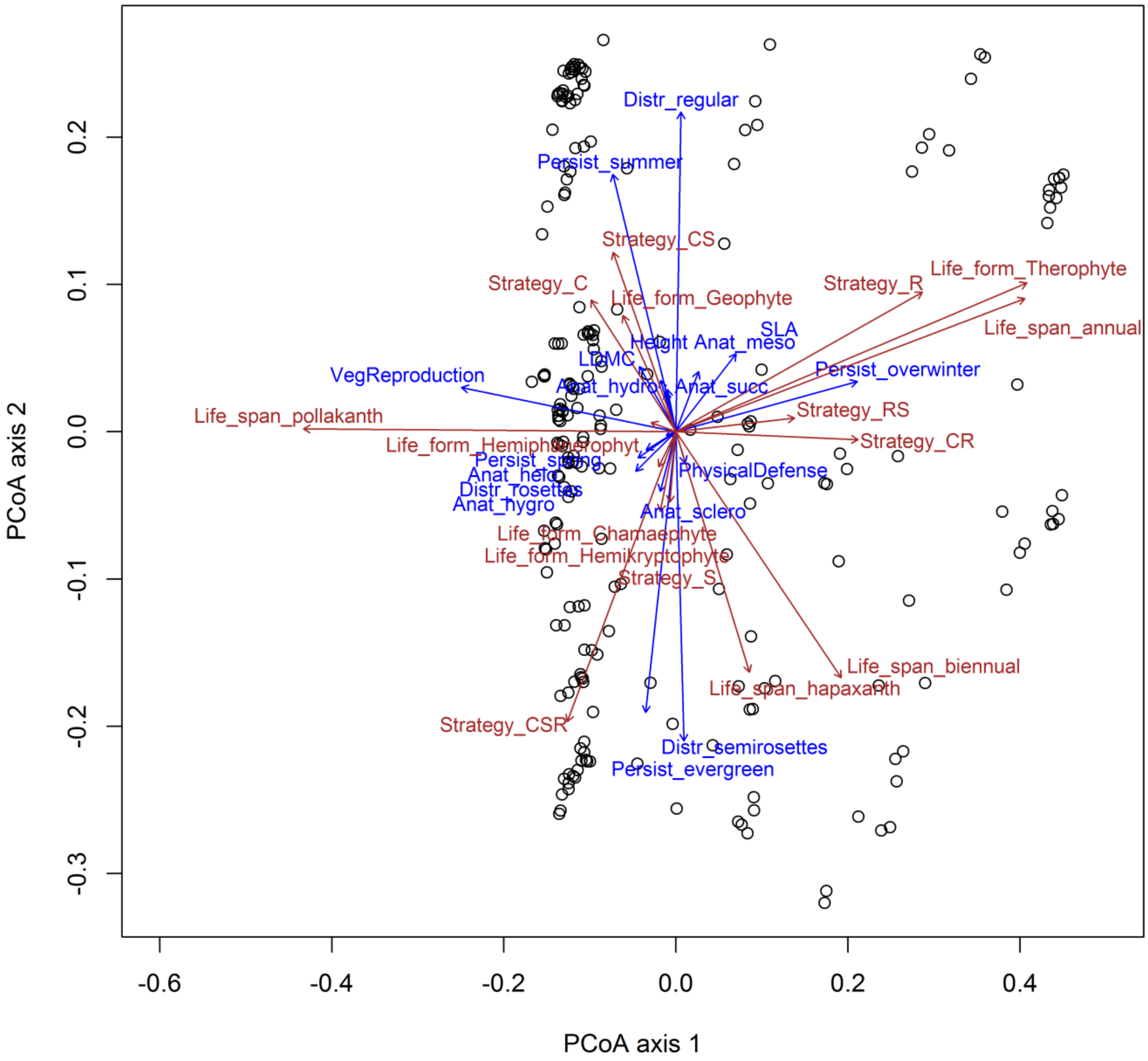

Figure S1: Principal coordinate analysis (PCoA) on multi-trait distances based on 11 traits (SLA, LDMC, height, anatomy, persistence, leaf distribution, vegetative reproduction, physical defense, life span, life form, strategy type) and all species, either planted as phytometers or resident species (n= 227). Traits included in the multi-trait distance used for selecting species in the different scenarios (see SI Table S1) are shown in blue, further traits are shown in brown. Trait correlations with the first and second PCoA axes were derived from a post-hoc regression. The further axes are captured by traits included in our multi-trait measure (3rd axis: leaf distribution, 4th axis leaf anatomy).

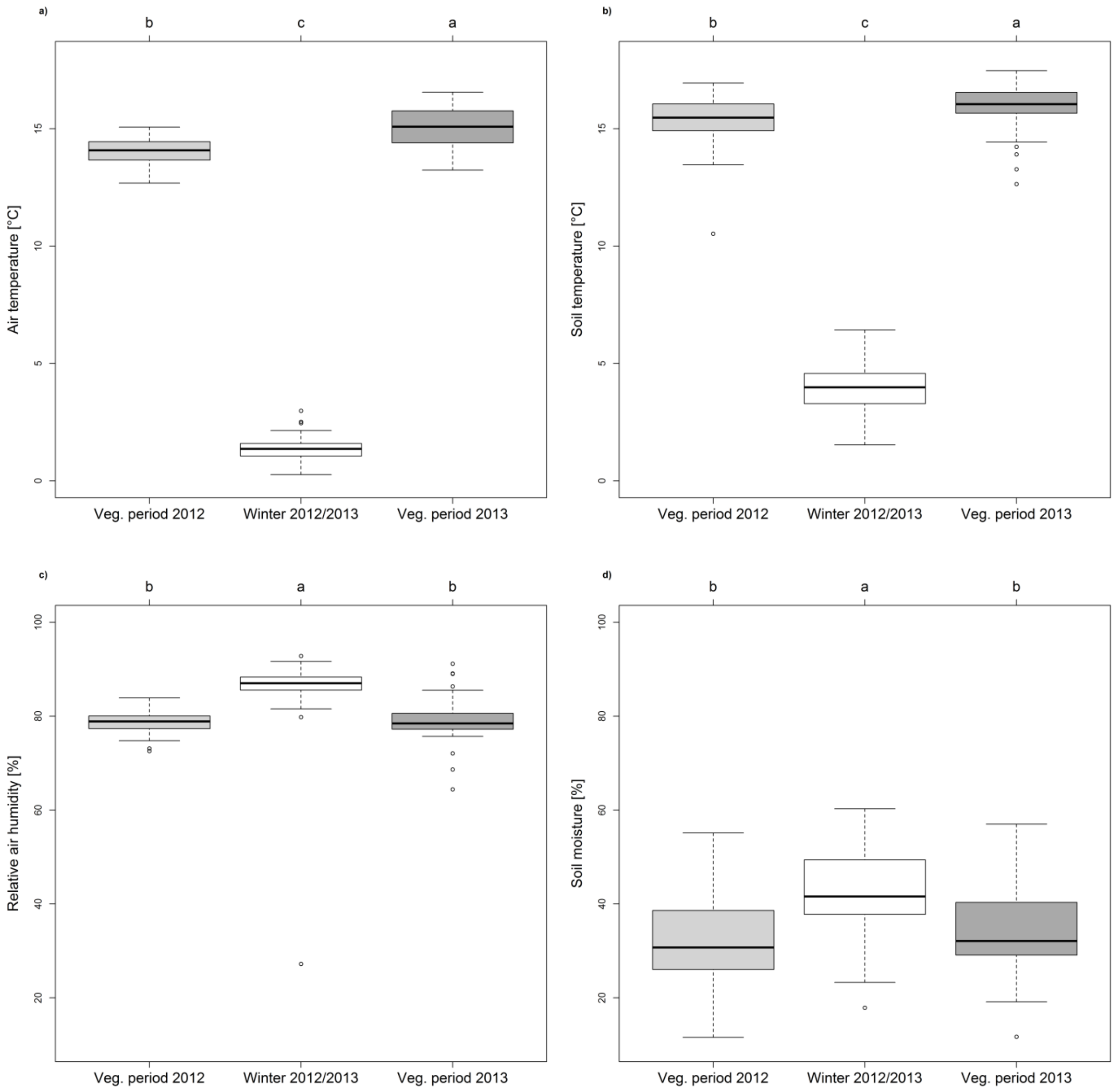

Figure S2: Mean climate data during the time of our experiment. Air temperature a) and relative air humidity c) were measured at two meters above ground level; soil temperature b) and soil moisture d) were measured at 10 cm below-ground. All climate data were measured at 10 minutes intervals by data loggers installed near the experimental plots and then aggregated to monthly mean values. Here, we present mean values aggregated to thethree investigated time spans (vegetation period 2012, winter 2012/2013 and vegetation period 2013) in our study plots (n= 54). Small letters refer to statistically significant differences according to a post-hoc Tukey test. Due to logger failure, in some plots the climate data were

not complete and plot number differed among time periods for the different variables (see SI Table S3).
